# A predictive chromatin architecture nexus regulates transcription and DNA damage repair

**DOI:** 10.1016/j.jbc.2025.108300

**Published:** 2025-02-11

**Authors:** Audesh Bhat, Sonali Bhan, Aindrila Kabiraj, Raj K. Pandita, Keneth S. Ramos, Sandhik Nandi, Shreya Sopori, Parthas S. Sarkar, Arti Dhar, Shruti Pandita, Rakesh Kumar, Chandrima Das, John A. Tainer, Tej K. Pandita

**Affiliations:** 1 Centre for Molecular Biology, Central University of Jammu, Jammu and Kashmir, India; 2 Biophysics and Structural Genomics Division, Saha Institute of Nuclear Physics, Kolkata, India; 3 Homi Bhabha National Institute, BARC Training School Complex, Mumbai, Maharashtra, India; 4 Center for Genomics and Precision Medicine, Texas A&M College of Medicine, Houston, Texas, USA; 5 Department of Neurobiology and Neurology, University of Texas Medical Branch, Galveston, Texas, USA; 6 Department of Pharmacy, Birla Institute of Technology and Sciences Pilani, Hyderabad Campus, Telangana, India; 7 ICON Clinical Research, San Antonio, Texas, USA; 8 Department of Biotechnology, Shri Mata Vaishnav Devi University, Katra, India; 9 Department of Molecular & Cellular Oncology and Department of Cancer Biology, UT MD Anderson Cancer Center, Houston, Texas, USA

**Keywords:** chromatin structure, transcription, DNA damage and repair, genomic instability

## Abstract

Genomes are blueprints of life essential for an organism's survival, propagation, and evolutionary adaptation. Eukaryotic genomes comprise of DNA, core histones, and several other nonhistone proteins, packaged into chromatin in the tiny confines of nucleus. Chromatin structural organization restricts transcription factors to access DNA, permitting binding only after specific chromatin remodeling events. The fundamental processes in living cells, including transcription, replication, repair, and recombination, are thus regulated by chromatin structure through ATP-dependent remodeling, histone variant incorporation, and various covalent histone modifications including phosphorylation, acetylation, and ubiquitination. These modifications, particularly involving histone variant H2AX, furthermore play crucial roles in DNA damage responses by enabling repair protein's access to damaged DNA. Chromatin also stabilizes the genome by regulating DNA repair mechanisms while suppressing damage from endogenous and exogenous sources. Environmental factors such as ionizing radiations induce DNA damage, and if repair is compromised, can lead to chromosomal abnormalities and gene amplifications as observed in several tumor types. Consequently, chromatin architecture controls the genome fidelity and activity: it orchestrates correct gene expression, genomic integrity, DNA repair, transcription, replication, and recombination. This review considers connecting chromatin organization to functional outcomes impacting transcription, DNA repair and genomic integrity as an emerging grand challenge for predictive molecular cell biology.

Life’s blueprint in DNA holds the information needed for the organism's survival and propagation. To protect this crucial cellular component and to accommodate it within the nucleus, most organisms have developed various strategies to compact their genomes into discrete structures known as chromatin. In the 1880s, Walther Flemming first observed a fibrous network in the nucleus and, noting its dye-absorbing properties, termed it chromatin, derived from the Greek word “chroma” (1).

Prokaryotes, lacking well-organized nuclei and histone proteins (except in Archaea), compact their genomes into membrane-less structures called nucleoids. This compaction is achieved through DNA supercoiling and the association with non-histone proteins (2). Bacterial DNA is typically negatively supercoiled during normal growth phases (3). Proteins such as the heat unstable (HU) DNA-binding protein and topoisomerase-I facilitate DNA condensation by creating sharp bends and turns, maintaining the supercoiling tension, and controlling transcription (4). Once the DNA is condensed, DNA gyrase and other proteins help sustain this supercoiling to maintain the supercoiled structure (5, 6).

In contrast, eukaryotes have evolved sophisticated mechanisms to package their DNA into chromatin within the double-membrane nucleus. These mechanisms also provide epigenetic marks established during development to maintain patterns of transcriptional regulation and persistent alterations in phenotype that may be reprogrammed in cancer (7). This structural organization is crucial not only for genome stability and packaging but also for regulating various molecular processes such as transcription, recombination, repair, centromere formation, and replication. Histone variants and post-translational modifications (PTMs) of histones are key in forming functional chromatin domains, directly influencing chromatin structure and, consequently, DNA-related activities. Disruptions in these assemblies can lead to chromatin organization defects, persistence of DNA damage, and compromised DNA processes, promoting genome instability (1, 8).

Chromatin organization thus plays comprehensive and pivotal roles in regulating DNA replication, gene expression, cell cycle progression, and DNA repair (9, 10, 11, 12). Recent findings suggest that various repetitive sequences in genome also influence gene expression (13). Genome stability is thus closely tied to its organization, with instability often arising from aberrant DNA conformation and replication, unchecked cell division, and DNA damage (14, 15). Chromatin plasticity in turn is crucial in responding to DNA damage and acts as a barrier against damaging agents (1), so it is a key determinant of both genome stability and biological outcomes. Here we therefore propose that delineating pairwise relationships of chromatin structure to patterns of gene expression and genomic stability suggests a path to prediction of outcomes for molecular cell biology. Notably, the 50-year-old grand challenge of protein structure prediction was solved by analogous pattern recognition to overcome otherwise insurmountable challenges from complex energy landscapes and conformational combinations (14, 15). This review considers established vital roles of chromatin structure in maintaining the gene expression and genomic stability key to life. We suggest that it will now be increasingly important for predictive biology and medicine to systematically build databases to determine pairwise relationship patterns that may address the emerging grand challenge of connecting chromatin organization to functional outcomes for DNA transcription, replication, repair, and genomic integrity.

## Complex chromatin organization of the genome

### Chromatin architecture at a glance

Our understanding of chromatin has evolved significantly over recent decades, shifting from a static structural view to recognizing chromatin as a dynamic and highly functional entity. In higher eukaryotes, chromatin forms various compact domains that function dynamically within the genome, although the specifics of these processes remain incompletely understood. Advances in live cell imaging, single nucleosome imaging, and the development of CRISPR/dCas9-based techniques have become valuable methodologies to make these ground-breaking discoveries to understand the chromatin functional dynamics (16, 17, 18).

In this section, we provide a concise description of the chromatin’s fundamental structure and focus more on the recent findings. For in-depth reading considerations, we recommend these excellent reviews (19, 20, 21, 22, 23, 24, 25, 26, 27, 28, 29, 30, 31, 32). Fundamentally, Our current understanding of the eukaryotic chromatin architecture is that a hierarchically ordered complex of DNA, histones, and non-histone proteins form an evolutionarily conserved process of packing DNA into a highly condensed structure (33). The nucleosome forms a fundamental unit of chromatin, consisting octamer (H2A, H2B, H3, and H4) around which 146 DNA base pairs are wrapped. These nucleosomes are connected by short linker-DNA segments bound to histone H1, giving the 10-nm chromatin fiber a “beads-on-a-string” appearance. The DNA-bound nucleosomes can then interact to form chromatin fibers (34).

The negative charge of DNA, due to its negatively charged phosphate backbone, is partially neutralized by the positively charged core histones. In the 10-nm fiber, there is a considerable electrostatic repulsion between the adjacent nucleosomes, especially in the absence of salt solution (35, 36). To achieve a higher-order chromatin folding, factors such as linker histone H1, Mg^2+^ cations, other positively charged molecules, and DNA-binding proteins like heterochromatin protein 1 (HP1) interact with chromatin (37, 38, 39, 40). In fact, charged interfaces are also key to DNA damage detection, excision specificity, repair pathway outcomes and even protein DNA mimicry to block protein-DNA interactions (41, 42, 43, 44). Such pervasive protein electrostatic interactions with DNA highlight the significant impact of the cellular distribution of electrical changes on chromatin organization and dynamics (35, 45, 46). The stability of secondary (30-nm fiber) and tertiary (>30-nm fiber) chromatin structures depends on DNA and histone modifications, histone variants, and architectural proteins such as methyl-CpG-binding protein 2 (MeCP2), HP1, and poly(ADP-ribose) polymerase 1 (PARP1).

Chromatin exists in either a relaxed, transcriptionally active state known as euchromatin or as a compact, transcriptionally inactive state known as heterochromatin, depending on the cell's functional status (47). Transcriptionally active euchromatin has a wider spacing between nucleosomes (48, 49), besides having the highest percentage of nucleosomes with precise locations in relation to the underlying DNA sequence (49), a feature considered important in transcription regulation near the transcription start sites (TSS) (50). The presence of euchromatin in transcriptionally active genomic regions, including promoter and enhancer regions, ensures access to transcription factors and chromatin remodelers that enable chromatin opening and transcription activation (51). Heterochromatin can be constitutive or facultative, with the former being consistently compact and enriched in repetitive, gene-poor sequences, and the latter capable of switching between active and inactive states (34, 52). Complex histone PTM patterns [Table 1], which specify various combinations of modifications and the proteins they associate with, inform the difference in chromatin structure. For example, pericentric constitutive heterochromatin is rich in histone H3 lysine 9 di/trimethylation (H3K9me2/3) and histone H4 lysine 20 trimethylation (H4K20me3) marks, whereas acetylation of H3K9 and H4 N-terminal lysines indicates a more relaxed chromatin state (53).

**Table 1:** List of histone PTMs for chromatin structure modulation and DDR

Histone	Residue	Enzyme	Modification	Proposed cellular function	Effect on chromatin compaction
H2A	Ser139	ATM, DNA-PKcs, ATR,	phosphorylation	DNA repair	Not known
	Lys119	BRCA1, RING2	ubiquitylation	Spermatogenesis	compaction
H3	Lys4	MLL, SETD7/9	methylation	transcriptional activation, permissive euchromatin (di-Me)	Not known
		SRC1, GCN5	acetylation	transcriptional activation, histone deposition	decompaction
	Lys9	Clr4, EHMT2, SETDB1, SU(VAR)3-9H1	methylation	genomic imprinting, DNA methylation (tri-Me), transcriptional activation, transcriptional silencing (tri-Me), transcriptional repression	compaction
	Lys27	EHMT2,EZH2	methylation	transcriptional silencing, X inactivation (tri-Me)	compaction
	Lys36	SETD2	methylation	transcriptional activation (elongation)	decompaction
H4	Lys5	ATF2, HPA2, p300 HAT, HAT1, TIP60	acetylation	histone deposition, transcriptional activation, DNA repair	decompaction
	Lys8	GCN5, PCAF, TIP60, ATF2, Elp3, p300 HAT	acetylation	transcriptional activation, DNA repair	decompaction
	Lys12	HAT1, TIP60, HPA2, p300 HAT	acetylation	histone deposition, telomeric silencing, transcriptional activation, DNA repair	decompaction
	Lys16	GCN5, TIP60, ATF2, Sas2	acetylation	Euchromatin, transcriptional activation, DNA repair	decompaction
	Lys20	SU(VAR)4-20H1, SU(VAR)4-20H2,SETD8	methylation	transcriptional activation/silencing, checkpoint response, 53BP1 loading following DSBs (di-Me), heterochromatin (tri-Me)	compaction

Besides being transcriptionally poor due to less accessibility to transcription factors and chromatin remodelers (54), heterochromatin is primarily located in the areas around the nucleolus and the nuclear periphery, where it is spatially separated from the euchromatin (55). The facultative heterochromatin typically assembles in areas with developmentally regulated genes that remain silent in response to developmental signals, whereas constitutive heterochromatin is found at the same genomic locations in all cell types and, once formed, spreads as an organism age progresses (56). In contrast to euchromatin, heterochromatin is primarily defined by sequence-independent epigenetic mechanisms and lacks nucleosome positioning (55). Histone tail methylation (H3K9me3)-related gene silencing underlies the biochemistry of heterochromatin formation by facilitating the binding of HP1 (57), which further dimerizes and connects adjacent chromatin fibers (58). Interestingly, recent studies have challenged the long-held notion that heterochromatization is exclusively dependent on chromatin compaction but rather is facilitated by phase-separation that involves demixing of HP1 in the liquid-liquid phase, creating liquid condensates around the heterochromatin (59, 60). A third state of the chromatin referred to as the Polycomb-repressed state was recently discovered in the *Drosophila* genome that has a distinct feature from the active and inactive states of the chromatin (61). This distinct state of highly compact DNA increased within domain intermixing, and a greater propensity for spatial exclusion of neighboring domains is believed to be predominantly governed by the Polycomb Group (PcG) proteins, a group of proteins known for their nucleosome bridging property (61, 62).

An important feature of chromatin is its dynamic nature. The dynamic nature controls diverse genomic processes, such as replication, transcription, and DNA repair/recombination *via* modifying DNA accessibility (63). With the advent of 3C (Chromosome conformation capture) technologies, especially the high throughput genomic and epigenomic (Hi-C) technology to study the 3D structure of the chromatin, researchers have been able to map the dynamic conformation of the genome more precisely up to kilobase scale. The first detailed conformation map of human genome build with Hi-C technique which was at megabase scale revealed the existence of two distinct genome wide compartments; open/active and closed/inactive, thus enabling spatial proximity of the gene-rich active regions within these compartments (64). Furthermore, advanced Hi-C mapping techniques helped to refine the mapping to kilobase scale, enabling the identification of another level of compartmentalization that involves the creation of self-interacting Topologically Associating Domains (TADs) (65, 66). Since TADs are present in a wide range of species and cell types, it is highly likely that they are a conserved and significant aspect of genome organization. However, their actual significance in regulating the expression of the genome is still unknown. More advanced techniques such as single cell nucleosome imaging and CRISPR/dCas9 are rapidly advancing the study of chromatin behavior in living cells. For instance, live cell Photoactivated Localization Microscopy (PALM) imaging of tightly chromatin-bound histone H2B (H2B-PA-mCherry) in HeLa cells has revealed that the chromatin exists in clusters or domains, with largely stable domain structure (radius ∼110 nm) across different phases of cell cycle (67, 68). Single molecule tracking of histone H2B has provided insights into chromatin mobility across different cell types. Additionally, the CRISPR/dCas9 approach is enhancing knowledge of 3D chromatin structure dynamics by allowing for the labelling and tracking of specific genome sequences in living cells (9, 18, 69, 70, 71).

To understand the influence of mechanical characteristics of DNA on chromatin organization on the mesoscale level, the construction of unprecedented 3D maps of nucleosome orientation and positioning was made possible through the development of advanced techniques, such as Hi-CO and Radiation-Induced Correlated Cleavage Sequencing (RICC-Seq) (72, 73). These approaches have led to the identification of chromatin folds on the tetranucleosome scale that are linked to or deficient at transcription start and finish sites, suggesting their functional relevance in transcription (74). Additionally, the latest findings suggest that chromatin possesses viscoelastic and nontrivial rheological characteristics, with various relaxation timelines and the ability to arrange into regions of variable mobility (75, 76). Specifically, the *in vivo* chromatin studies have revealed solid-like behavior and structural relaxation that happens on an hourly basis, like the pace of enzyme-mediated histone changes (77, 78). In yet another milestone study, the influence of various epigenetic modifications on chromatin packaging recorded at the kilobase-to-megabase scale revealed a distinct pattern defined by the nature of the modification (61). Future research, combining Hi-CO or RICC-seq data with the sequence dependence of DNA bendability and torsional rigidity is likely to demonstrate how sequence-encoded DNA mechanical properties can support the necessary twists and bends of linker DNA to achieve particular functional 3D nucleosome orientations (74). Sequence-encoded non-B DNA structures such as G-quadruplex can also promote open chromatin, transcription, and instability. For example, a comprehensive genome-wide analysis identified G-quadruplex enrichment at transcription start sites in human, but not in chimpanzee and mouse genomes, with the most mutagenic G-quadruplex mapped to regions promoting alternative DNA structures (79).

### Organization of chromatin at the centromeric region

Centromeres are the unique genomic sites where spindle fibers bind during cell division to pull chromosomes toward the opposite poles. Even though eukaryotes share a high degree of conservation in their overall chromosome segregation machinery, the DNA and protein components of the centromeric region show substantial divergence (80). Centromeric nucleotide sequence varies in length from a point centromere of ∼100 bps in budding yeast to several megabases long in most plants and animals, including humans, and to a whole chromosome in some flowering plants, insects, and nematodes, and it is highly repetitive with long arrays of tandem repeat known as satellites (80, 81). Highly specialized centromeric chromatin is distinguished by the presence of nucleosomes, where histone H3 is replaced by its variant Centromere protein-A (CENP-A), which serves as the basis for the formation of kinetochore (82).

The centromeric drive model explains the rapid evolution of centromeric DNA and essential centromere proteins by arguing that through asymmetric meiosis, in which one meiotic product becomes the oocyte nucleus and the other three are lost as polar bodies, centromeric DNA may function as a selfish genetic factor and promote non-Mendelian segregation. Centromeric proteins are selectively pressured to adapt by suppressing the resulting fitness costs, which raises genetic conflict with the rest of the genome (80). It has been suggested that CENP-A functions as the suppressor of centromere drives in males with symmetric meiosis by accumulating mutations that stabilize its interactions with centromeric DNA variations, hence reducing the detrimental effect of centromere drive (83). A CENP-A-specific histone chaperone known as HJURP attracts CENP-A to centromeres. Although HJURP was first identified as a protein that binds cruciform structures, HJURP can evidently identify centromeric satellites by looking for DNA structures that are projected to be enriched on the satellite centromeres and neocentromeres (84).

Although centromeric maps of human centromeres have recently been created, there is more to be done to use these maps for functional research (85). Large pools of unassembled centromeric α-satellites have been *de novo* isolated and classified using a bottom-up functional genomics-based method based on CENP-A binding (86). The resulting CENP-A chromatin complex is positioned on α-satellite dimers with distinct footprints, and changes in the footprints of the CENP-A chromatin complex are linked to sequence variations in α-satellite arrays, indicating that CENP-A assembly is driven by a sequence-dependent genetic component (87).

#### Epigenetic landscape of centromeres

H3K4me2 is an attribute of chromatin in the centromere core that is typically linked to active chromatin and intermingled with CENP-A (88). The physiological significance of centromere H3K4me2 lies in its necessity for CENP-A assembly on a synthetic human kinetochore and HJURP targeting (89). Centromere integrity is dependent on H2B mono-ubiquitination, another alteration linked to actively transcribed chromatin. Its loss results in heterochromatinization of centromere cores and inadequate/inappropriate chromosomal segregation (90). The distribution of H3K4me2 is not uniform across the centromere. The abundance of H3K9me3 and less of H3K4me2 is also observed at the centromeric core (91). This heterogeneity is critical for maintaining the centromere (92). The histone acetyltransferase KAT7/HBO1/MYST2 is important to prevent heterochromatinization at the centromere (93). It limits H3K9me3 build-up and centromere shutdown by encouraging nucleosome turnover and is linked to Mis18, a component of the CENP-A loading machinery, in the centromere core in the G1 phase.

#### Centromeric nucleosomes structure

The CENP-A-containing octamers are structurally different than that of H3-containing octamers. CENP-A loop 1 reveals two additional amino acid residues (Arg 80 and Gly 81) extending from the CENP-A nucleosome. The way DNA is wrapped around the nucleosome that contains CENP-A is impacted by the fact that the αN helix of CENP-A is one turn shorter than that of H3 (94, 95). This decreases the number of DNA interaction sites, making DNA more flexible at the locations where nucleosomes enter and depart (96, 97).

Based on investigations employing chimeras of H3 and CENP-A, loop 1 and the α2 helix form the primary CENP-A region that regulates centromere positioning and function. This has also been known as the CENP-A targeting domain (CATD) (98, 99). The CATD is also necessary to direct CENP-A to the centromere by forming an association between human CENP-A and its histone chaperone HJURP (100). Both the human and *S. cerevisiae* HJURP/CENP-A/H4 heterotrimers' crystal structures showed that the CATD connects with the N-terminal of HJURP, while HJURP's C-terminal limits the CENP-A/H4 heterodimer's DNA-association, averting the assembly of a (CENP-A/H4)2 tetramer and early DNA association (101, 102).

More recent FRET-based studies on the dynamicity of the centromeric region reveal chromatin structure as being made open and extremely dynamic by CENP-A (103). This study further showed even though chromatin with CENP-A can form a more complex structure, the dynamicity of the CENP-A nucleosome causes nucleosome stacking interactions to be momentary and thus, significantly improving fiber access. Recent cryoEM-based studies couldn’t score stabilization of CEBP-B *via* CENP-A mediated interactions in the nucleosomes. However, cryoEM results suggested that CENP-B may be further involved in opening chromatin structure, thus affecting DNA unwrapping (103). Going forward, combining computation with experimental methods such as cryoEM and X-ray scattering to define and even target dynamicity (104, 105) is expected to be critical to predictive knowledge for chromatin structure function relationships.

### Organization of chromatin at the telomeric region

Located at the ends of chromosomes, telomeres are specialized nucleoprotein structures, consisting of repetitive hexanucleotide sequences, (TTAGGG)_n_ in humans. Telomere sequences terminate in a short single-stranded G-rich 3′ overhang (50–500 nucleotides in humans) but are primarily double-stranded (10–15 kb in humans) (106). The organization of chromatin and nucleosomes at telomeres is crucial for numerous physiological functions, including genomic stability, the protection of and maintenance of telomere ends. Telomeric chromatin prevents unnecessary recognition and activation of the double-strand break (DSB) repair system and mediates telomeric elongation. This protective role is facilitated by interactions with various protein complexes, such as telomerase and the Shelterin complex (107).

**Figure 1:**
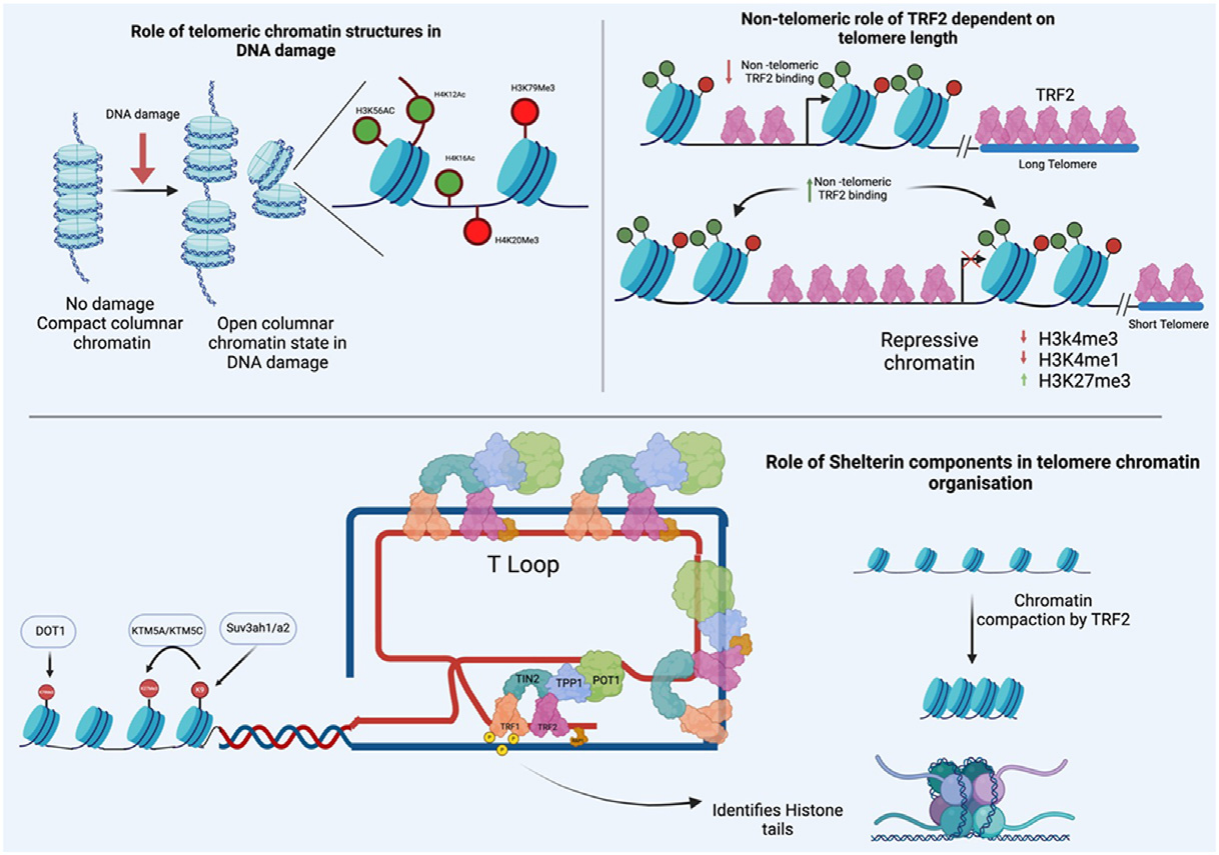
**Interplay between the epigenetic landscape of telomere and Shelterin complex in mediating telomeric and non-telomeric functions in chromatin organization.** The Shelterin complex is important in orchestrating telomere biology. It mediates telomeric chromatin organization *via* its components TRF1 & TRF2. Besides, higher-order columnar chromatin organization at the telomere opens at right angles upon DNA damage. This exposes several histone modifications like H3K56, H4K12, and H4K16 acetylation and H3K9, H3K79, and H4K20 methylation key in mediating DNA damage-related responses. Current studies are identifying non-telomeric functions of Shelterin-component TRF2, which is recruited at non-telomeric promoter regions, highlighting the role of TRF2 in non-telomeric functions.

Shelterin, a six-subunit protein complex of proteins including (TRF1, TRF2, POT1, TIN2, TPP1, and RAP1), is essential for telomere-related functions and maintenance. Components TRF1 and TRF2 bind to the double-stranded regions of the telomere (108), and POT1 binds to the single-stranded telomere sequences. TPP1 interacts with POT1: it stabilizes its interaction at the telomeric region and recruits telomerase to the telomere. TIN2 acts as a connecting element between TRF1-TRF2 and TPP1-POT1 (Fig. 1). Shelterin components prevent inappropriate chromosomal end maintenance to DNA damage by inhibiting Ataxia-Telangiectasia mutated (ATM) and ATM- and Rad3-related (ATR) (106). Contribution of the Shelterin complex in telomeric biology is substantial, protecting telomeres from aberrant activation of DNA damage response (DDR) in mediating appropriate elongation of the telomeric sequences.

#### Epigenetic landscape of telomeric and sub-telomeric region

The epigenetic landscape at telomeres encompasses a complex interplay between chromatin structures, DNA and histone modifications, and non-coding RNA molecules all of which regulate telomere maintenance and function. Telomeres, as chromatin structures, are subject to epigenetic regulation (including DNA methylation and histone modifications) and also act as epigenetic regulatory elements influencing chromosomal stability (109). Across species, telomeric and sub-telomeric regions typically exhibit features of repressive chromatin and changes in this environment can lead to telomere-length dysregulation and increased recombination (110). Consequently, telomeric regions are generally hypoacetylated.

Sirtuin 6 (SIRT6), a member of the silencing information regulator 2 (Sir2) family, is a deacetylase in mammals, deacetylating residues at H3K9, H3K18, and H3K56 (111, 112). SIRT6 functions primarily at telomeres and plays a crucial role in preventing telomere defects (113). Several histone methylation marks (such as H3K9me3, H3K27me3, H3K79me3, and H4K20me3) invoke repression and promote a heterochromatin state (Fig. 1). Mouse knockout studies demonstrated an abundance of H3K9me3 (a heterochromatic mark) in telomeric areas, contributed by histone methyltransferases Suv39h1 and Suv39h2 (also known as KMT1A and KMT1B). Furthermore, H3K9me3 leads to HP1 localization at telomeres (114), which recruits KMT5B and KMT5C methyltransferases to establish H4K20me3, another heterochromatic mark at the telomeres. Additionally, H3K79me3, mediated by Dot1L, contributes to the formation of H4K20me3 (115) (Fig. 1). An interesting model suggests that SUV39H1, SUV39H2, and DOT1L initiate a first wave of telomeric heterochromatinization, which is further amplified by HP1, KMT5B, and KMT5C.

Ongoing research is identifying euchromatic features within telomeric regions. For example, H3K9me3 is concentrated in telomeres in mouse embryonic stem cells and installed by SET domain bifurcated histone lysine methyltransferase 1 (SETDB1) rather than SUV39H1/2. This discovery is intriguing since SETDB1 is primarily associated with euchromatic regions (116). Chromatin immunoprecipitation (ChIP)-sequencing studies in human T cells reveal a decrease in H3K9me3 in telomeres, with significant enrichment in sub-telomeric areas (117). The same research identified significant telomeric enrichment of two activating markers, H2BK5me1 and H3K4me3.

Although uncertainty remains regarding the complete epigenetic landscape of telomeres, sub-telomeric regions are predominantly heterochromatic (117, 118). High levels of repressive marks like H3K9me3 and H3K27me3 are observed in these regions. Notably, while some studies have identified a hypoacetylated status of sub-telomeres (118), other studies, such as one by Rosenfeld and co-workers (117), show an abundance of acetylation marks indicative of activation, namely, H2AK5ac and H3K14ac.

In budding yeast, H2BK123 ubiquitination by Rad6 and Bre1 is essential for the formation of other histone PTMs like H3K4me and H3K79me (119, 120, 121), which acts in establishing a heterochromatin landscape. H2BK123ub also contributes to telomere replication. The Spt-Ada-Gcn5 acetyltransferase (SAGA) complex (including its deubiquitinating module) deubiquitinates H2BK123 (122). Loss of Sus1, part of the SAGA complex, leads to increased H2BK123ub and facilitates telomere extension, underscoring the importance of this modification in maintaining telomere homeostasis.

#### Telomeric nucleosomal structure

Telomeric nucleosomes exhibit unique structural features compared to bulk of the bulk chromatin. Telomeric DNA is organized into compact nucleosome structures with shorter linker DNA (123, 124). Nuclease digestion studies show that telomeric nucleosomes are separated by linker DNA about 40 bp shorter than that found elsewhere. These unusual properties—such as nucleosome spacing and lack of positioning—are essential for telomere function. Subsequent studies show that while the specific telomeric sequence is crucial in organizing telomeric chromatin, it is not the sole contributor.

Recent structural studies have reveal specialties of telomeric chromatin. Telomeric nucleosomes are less stable in solution, with more unwrapped DNA along the nucleosome core particles (NCPs) conferring dynamicity to telomeric nucleosomes. A recent study with correlative light and electron microscopy showed that telomeres are arranged in a fibrous mesh-like structure within cells (125). Further cryo-EM studies show that telomeric chromatin can form higher-order columnar structures using tetra-nucleosomes in the presence of Mg2+ (126). The H2A C-terminal tail acts in forming these structures. Under DDR conditions, an open state of the columnar structure state is observed, where two constituent nucleosomes are at right angles to each other. The contact region between two stacked nucleosomes is where many epigenetic modifications, including methylated H3K9, H3K79, and H4K20 and acetylated H3K56, H4K12, and H4K16, are strategically situated and exposed (Fig. 1). This observation suggests that chromatin dynamics at the telomere are structurally influenced by PTMs at the histone tail.

#### Role of Shelterin in telomeric chromatin organization

The Shelterin complex plays a crucial role in organizing and maintaining the chromatin structure in telomeric regions, but its mechanism of interaction with telomeric chromatin and nucleosomes is complex and poorly understood. The Shelterin component TRF1 can identify telomeric interaction sites within nucleosomes, forming a ternary complex (127) (Fig. 1). TRF1-induced changes in telomeric nucleosomes occur without detachment of histone subunits, suggesting a role in telomere capping functions. Atomic force microscopy (AFM) imaging showed that TRF1 leads to telomeric DNA compaction, which occurs in the presence of nucleosomes, indicating TRF1-nucleosomal interactions (128). This remodeling TRF1 activity highlights its binding properties in the chromatin context, offering insights into chromatin dynamics.

Cryo-EM results show that the Myb2 domain of TRF1 binds H3 and H2A, resulting in a shift of 1 bp (129). To mimic a more physiological environment, studies using EMSA-based assays revealed interactions between telomeric subcomplexes containing TRF1-TIN2-TPP1. Residues 431 to 439 at the C-terminus of TRF1's Myb2 domain acted in this process, binding basic residues of the H3 tail and displacing phosphate groups. Mutational experiments identified phosphorylation of residues Ser434, Ser435, and Ser437 of TRF1 in these interactions. Although the kinases for Ser434 and Ser437 phosphorylation are unknown, polo like kinase (PLK1) phosphorylates Ser435, catalyzing TRF1's interaction with telomeres (130) (Fig. 1). TRF1 installs Blooms (BLM) RecQ like helicase to promote replication ahead of the G-quadruplex structure, and TRF1-deficient cells show severe replication defects compared to BLM-deficient cells (131, 132). Interestingly, BLM is an ancient gene often overexpressed in cancer cells that leads towards a unicellular hyperproliferative state that is associated with poor survival (133). These findings link TRF1 and BLM, and suggest that disassembly of telomeric nucleosomes by TRF1 facilitates replication.

TRF2 is another essential Shelterin complex component that acts in telomeric chromatin organization. Overexpression studies in mice and humans show that TRF2 down-regulates nucleosome occupancy and increases nucleosome spacing (134, 135). AFM data suggest that TRF2 binding compresses nucleosome fibers (136). Electron microscopic analyses show that both the full-length TRF2 and N-terminal truncated TRF2 induces the formation of columnar nucleosomal structures specific to telomeres (137). Single-molecule force microscopy further revealed that TRF2 binding stabilizes these columnar structures. Observations of native chromatin at telomeres indicate that the fiber width tagged with TRF2 (15.4 nm) is slightly larger than that tagged with H2B (14.4 nm), suggesting that TRF2 is attached to the telomeric chromatin surface, increasing fiber diameter. In the presence of TRF2ΔN, a similar mean fiber diameter of 15.3 nm was observed, indicating that TRF2 interacts with telomeric chromatin by contacting major and minor grooves across several nucleosomes. This columnar stacking, strengthened by histone tail elements, has significant implications for telomere structure and function, as shown by recent cryo-EM structures (126).

Current research is expanding knowledge of Shelterin to include non-telomeric functions and non-canonical roles in sub-telomeric regions. For instance, RAP1, a component of Shelterin, plays a role in controlling nuclear factor-κB (NF-κB) signaling. Upon NF-κB activation at the plasma membrane, cytoplasmic RAP1 forms a complex with IκB kinases (IKKs), leading to p65 phosphorylation, which enables NF-κB to bind proteins that remodel chromatin. The *TerF2IP* gene, which encodes RAP1, is modulated by phosphorylated p65 after its transport to the nucleus (138). Despite the critical role played by the Shelterin complex in telomere protection, the mechanisms by which it interacts with telomeric chromatin and nucleosomes remain elusive.

Moreover, recent studies reveal the promoter occupancy of TRF2 at non-telomeric sequences. Such occupancy was observed in cells with short telomeric lengths. In cells with longer telomeres, telomeric TRF2 binding was increased, such that its extra-telomeric occupancy at non-telomeric sites was reduced compared to cells with short telomeres (139). A telomere sequestration and partition model suggests that the binding of TRF2 at either telomeric or non-telomeric regions depends on the telomere length. Additionally, TRF2-target promoters exhibited signs of a changed epigenetic state, such as the presence of the histone activation (H3K4Me1 and H3K4Me3) and suppression (H3K27Me3) marks (139).

## Maintenance of the genomic integrity at distinct sites of the genome

### Heterochromatic repetitive sequences show genomic instability

In most eukaryotes, constitutive heterochromatin is enriched with repetitive sequences, primarily found in pericentromeric and telomeric regions, which define key genomic features (140). Besides these regions, repetitive sequences are also key features of micro- and mini-satellites, centromeres, and transposable elements (TEs). Typically, these regions are devoid of genes and have nucleotide repeat lengths ranging from five to a few hundred base pairs (141). To maintain genomic integrity, it is crucial to structurally and functionally regulate DNA repeats and TEs. Repeated DNA sequences are hotspots for meiotic crossover and other recombination events, replication errors, and DSBs, all of which can contribute to genomic instability (142). Recombination between repeated DNA sequences often leads to chromosomal rearrangements, a hallmark of cancer and many human genetic disorders (141, 143). To mitigate unauthorized recombination among dispersed repeating DNA regions, heterochromatin formation at these elements has evolved as a protective mechanism to reduce the possible occurrence of aberrant recombination events (141).

H3K9 methylation at pericentric heterochromatin disrupts the suppressor of variegation 3-9 homolog 1/2 (SU(VAR)3-9H1/2), which severely reduces cellular viability and induces chromosomal instability (144). Notably highly repetitive rDNA is related to heterochromatin, where reduction of histone H3K9me2/3 and related SU(VAR)3-9 in *drosophila* results in nucleolar disruption and an aberrant recombination that increases the formation of extrachromosomal circular DNA (145). The formation of complex DNA structures may be suppressed by heterochromatin linked to rDNA repeats to preserve genome stability by reducing recombination aberrations. Even as pericentromeric heterochromatin decompaction and transcription activation are documented in various hereditary disorders, such abnormal activation has also been seen in lung cancer (146, 147). A decrease in the condensed sections of the genome and loss of H2AK119Ub at satellite repeats are linked to the loss of BRCA1 (148). Similarly, due to loss of lysine-specific demethylase 2A (KDM2A), transcriptional activation and decompaction of heterochromatic elements happens, causing genomic instability and chromosome segregation defects (1, 149, 150). Derepression of pericentromeric satellite DNA results in increased DSBs, mitotic abnormalities, and a general loss of heterochromatin integrity (148).

Besides repetitive sequences, TEs have an inherent property to move within the genome. Thus, the TEs can cause genomic instability and alter gene structure and function by inserting themselves into coding sequences or regulatory regions of genes (141). Therefore, to prevent the unfettered mobility of TEs across the genome, an evolutionary strategy involving heterochromatin-mediated TE silencing may play a major role. This preventive process primarily involves three epigenetic processes: DNA methylation, H3K9 methylation, and the P-element-induced wimpy testis in *Drosophila* pathways. These molecular processes are active mostly during the developmental stages (141).

Heterochromatic structures must be reliably copied to preserve genomic stability and to prevent unauthorized recombination between the enormous amounts of repetitive DNA in the genome. In this regard, heterochromatin late replicating domains are efficiently replicated by several of chromatin-remodeling factors, such as switch/sucrose non-fermentable (SWI/SNF), imitation SWI/SNF, chromodomain helicase DNA binding, and inositol requiring complex family proteins, more likely through chromatin remodeling, thus allowing the replication fork to progress smoothly (151). Additionally, the SWI/SNF family of nucleosome remodeler SMARCAD1 (SWI/SNF-related, matrix-associated actin-dependent regulator of chromatin, subfamily A, containing DEAD/H box1) facilitates the formation of repressive and compact chromatin at the heterochromatic regions soon after its replication, by promoting deacetylation of the newly deposited histones by replicative chaperones (152). SMARCAD1 is also required for resection during DNA DSB repair (153). Moreover, nucleosome remodeling and deacetylase complex promotes genome stability by safeguarding higher-order chromatin structure, and upon the loss of any nucleosome remodeling and deacetylase complex components, such as HDAC1, RBBP4, and RBBP7, the maintenance of histone modifications and higher order chromatin structure is compromised (1, 154). These data indicate that loss of heterochromatin structure and impaired DNA replication is responsible for loss of genome stability, making chromatin more prone to DNA damage (1). Conversely, impaired DNA replication in late-replicating heterochromatic regions can contribute to genomic instability (140) and activate inflammation and immunity (155).

### Transcription and replication regulation by chromatin structure maintains genomic stability

Based on initial studies on chromatin organization, chromatin was thought to have various roles other than DNA compaction and packaging. Further studies revealed that nucleosomes physically obstruct *in vitro* transcription, and it was observed that deletion of histone N-terminal tails is responsible for change in gene expression (156, 157). Eukaryotic chromatin organization has profound involvement in transcription activation as compared to prokaryotes (1). In prokaryotes, the regulation frequently takes place by the binding of transcription factors near or at the promoter sequences which can further improve or block binding of RNA polymerase (158). Additionally, the role of promoter sequence in the transcription activation is characterized in *Escherichia coli,* and the association between gene expression, transcriptional factor (TF) binding with various regulatory sequences has been mapped. In eukaryotes, there are defined promoter sequences as well as enhancer sequences for the binding of general or gene specific TFs or co-activators at cis-regulatory elements for precise and temporal regulation of gene expression.

DNA replication often works in coordination with transcription, chromatin remodeling factors, epigenetic modifications, and repair processes, to ensure a faithful duplication of the epigenetic and genetic characteristics of the chromatin and maintenance of genomic stability. Replicating genomic DNA is a complex task. Although it is highly susceptible to errors, most eukaryotic cells must replicate millions of base pairs in a timely fashion, and yet eukaryotic cells manage to achieve this feat with a bare minimum of base-pair alterations. To do this, cells regulate DNA replication in a spatiotemporal manner to ensure a serial and accurate replication of the genome. Any deviation in the replication timing can interrupt the spatiotemporal segregation programs of the early and late replicating domains (1). To maintain an adequate level of histones, while avoiding aggregation of excess histones, regulatory mechanisms must be present in the transcriptional process. For regulating the stability, mammalian histone mRNAs have stem-loop organization at the 3′ end instead of a polyA tail (159). To enforce replication origin specificity, nucleosomes block non-specific loading of minichromosome maintenance helicase, as seen in the *in vitro* chromatin replication assays (160, 161). Abnormal replication licensing factor loading and initiation of uncontrolled replication happens due to loss of chromatin compaction control, which can lead to loss of genome integrity (162). Yet, when head-on transcription-replication conflicts (TRC) do occur, DNA repair proteins such as BRCA2 reduce resulting genomic instability (163).

Current evidence suggests that replication and transcription occurring at different time points during S-phase is perhaps a straightforward means to restrict TRCs through mutually exclusive chromatin occupancy. High-resolution live-cell imaging studies reveal a global anti-correlation between transcriptionally elongating RNA PolI and PCNA, a key replisome component (164). Additional evidence in support of spatiotemporal isolation of transcription and replication as a general mechanism to limit TRCs came from the sequencing of nascent transcripts in S-phase, showing that the periodically transcribed genes in early-replicating regions are transcribed late and *vice versa* (165). However, early genome-wide studies proposed that transcriptional units are the preferred location for mammalian replication origins, and this preference is expression-dependent (166). Also, a recent genome-wide replication-origin mapping study has reliably demonstrated that genes with high transcriptional activity have early-firing origins at or close to the TSS (167). Various studies have revealed the association between increased transcriptional activity and endogenous replication origins in human cells (167, 168). Despite recent advancements, it remains unclear how exactly TRCs are resolved and managed as well as how aberrant TRCs lead to genomic instability.

The plasticity of chromatin provides a dynamic binding platform for regulatory proteins and environmental conditions suitable for the regulation of gene expression. This predominantly entails chromatin remodeling, histone modifications, and histone turnover. Transcription involves three steps: initiation, elongation, and termination. Histone chaperones and remodeling complexes likely work in coordination to advance transcription *via* the chromatin template (169). Such coordination also helps histones to redeposit onto transcribed regions. Disturbance during histone redeposition will leave DNA exposed to transcription factors, allowing their binding to cryptic promoters (170), therefore causing wide-spread transcription and excessive R-loop (DNA–RNA hybrids) formation. This can cause genomic instability and replicative stress by blocking the processes of replication and transcription processes (171). The unpaired ssDNA of the R-loops is prone to breaks by nucleases, exogenous genotoxic stress and activation-induced cytidine deaminase induced genomic instability (172). Additionally, the high propensity of deoxycytidine to deoxyuridine conversion in the unpaired ssDNA of the R-loop increases the susceptibility for DSBs and recombination (173). Post- transcription termination step, deacetylation and redeposition of histones are required for maintaining chromatin stability within the genic regions. During S-phase, highly transcribing genes have an increased chance of conflicts with the replication fork, thus they are a potential threat to genomic stability (174). As transcription and replication mechanisms have a common template, evidences suggests that their collision happens, causing genomic instability (174). Additionally, collision of RNA and DNA polymerases show a high possibility of inversions, deletions, translocations and duplications (175).

#### Chromatin structural role in the repair of DNA damage induced by endogenous genotoxins

In human cells, mutations caused by endogenous genotoxic agents account for most of the genomic changes (176, 177). DNA damage is largely caused by endogenous reactive oxygen species (ROS), methylating/demethylating agents, hydrolytic deamination, and carbonyl stress (178). In an environment where DNA repair is competent, unrepaired endogenous damage can still occur because genotoxic stress generated from normal cellular activities *e.g.*, transcription and replication can exceed the high-fidelity DNA repair capacity of normal cells (176, 179). Majority of the DNA lesions caused by endogenous agents (∼75%) are single-strand DNA breaks (SSBs), which can transform into DSBs during replication (180, 181). The question of rapid and accurate DSB repair mainly arises when the genome is exposed to various physical or chemical agents such as radiomimetic drugs and ionizing radiations. Despite being less common, DSBs are much more hazardous and challenging to repair due to a complete physical break of the DNA backbone (182). To signal repair, ATM at the site of DNA damage undergoes autophosphorylation and dissociates from its inactive dimeric state to its active monomer state (177, 183, 184). ATM also happens to be a part of the DSB detection apparatus during meiosis, mitosis and the free radical induced breaks (182, 185, 186). Moreover, hierarchical signaling networks act at DSB responses and repair that coordinate chromatin structural alterations, particularly histone modifications. These affect cell-cycle checkpoint mechanisms through metabolic pathways to repair the damaged DNA ends (8, 187). Acetylation, methylation, phosphorylation and ubiquitylation are the exemplary histone modifications that play critical roles in executing efficient and flawless DNA damage repair (8, 12, 188, 189, 190).

Defective DSB repair and DDR to ionizing radiations (IRs) are correlated with lower levels of histone H4 acetylation at lysine 16 (H4K16ac). Acetylation of H4K16 is regulated by lysine acetyl transferase MOF (Males absent On the First) protein encoded by the *MOF* gene (191, 192). IR-induced DDR is prevented by depleting MOF which in turn prevents H4K16ac deacetylation (192). The DNA-dependent protein kinase catalytic subunit (DNA-PKcs), which initiates DSB repair by nonhomologous end-joining (NHEJ), is connected to MOF (193). In MOF-depleted cells, ATM-dependent IR-induced phosphorylation of DNA-PKcs was abolished, suggesting that MOF is required for the phosphorylation-dependent activation of DNA-PKcs and DSB repair processes. According to published data, depletion of MOF significantly reduces both homologous recombination (HR) and NHEJ-mediated DSB repair. By modifying histones at H4K16ac sites, MOF plays a crucial role in initiating appropriate cellular DDR to elicit DSB repair at multiple stages (192, 194, 195). MOF as well as TIP60, another key histone acetyl transferase function during DNA damage repair and transcription, thus reveal an interplay among histone modifications in transcription and DNA damage repair to maintain genome integrity (187, 196, 197).

Cells rely on DNA damage-specific repair processes to maintain genome stability and integrity. The repair pathways *e.g.*, mismatch repair (MMR), base excision repair (BER), and nucleotide excision repair (NER) resolve the nucleotide mismatch, single base alteration, and alterations involving nucleotide dimers or intra- or inter-strand cross-links, respectively. The single strand break repair (SSBR) mechanism resolves the SSBs whereas the NHEJ, alternative NHEJ (alt-NHEJ), and HR pathways repair the DSBs (198, 199, 200). The choice of rapid error-prone NHEJ, minor alternative-end joining, or the slower error-free HR pathway depends on the cellular context, including cell cycle phase (14, 193, 200, 201, 202, 203). Experimental evidence suggests that during NER, chromatin needs to be made readily accessible to various repair proteins for efficient and accurate damage detection and repair. Subsequently the chromatin architecture must be restored to reclaim genomic stability and possibly to preserve epigenetic landscape or to leave an imprint of the damage on the chromatin (204, 205).

Genomic alterations are greatly influenced by the presence of heterochromatin and euchromatin-like domains (206). Structural chromatin organization of a key determinant of local mutation rate in cancer cells, and DNA base substitutions are found more commonly in the heterochromatic regions and late replicating domains compared to the open, early replicating chromatin (12, 207). Therefore, the higher rate of somatic mutations strongly correlates with the heterochromatin-associated PTMs, such as H3K9me3/2 and H4K20me3, than with the euchromatin-associated genetic and epigenetic markers, such as histone H3 and H4 N-terminal tail acetylation, H3K4/K36me3 and GC content (206). These observations suggest that the chromatin architectural landscape impacts both the DNA lesion formation and repair effectiveness. Multiple causes are likely at play, such as chromatin accessibility to DNA repair machinery, variations in the capacity of signal repair, and elevated mutagen exposure at the nuclear periphery besides possible sequence-specific variances in mutation rates (208, 209).

**Figure 2:**
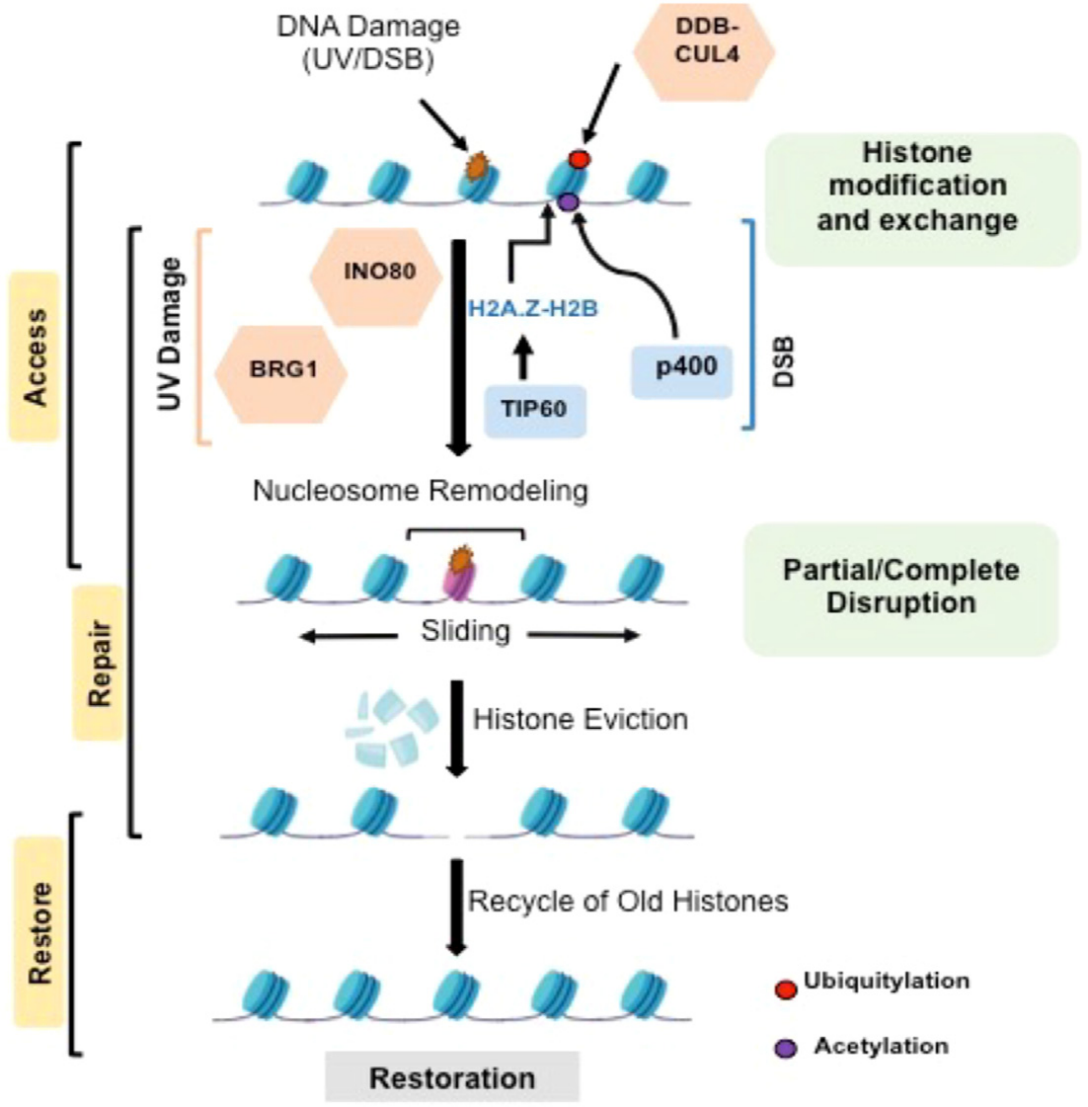
**Structure of chromatin in DNA damaged condition to establish the access-repair-restore model.** DNA-damage-induced histone modifications by acetylation (Ac) and ubiquitylation (Ub) promote nucleosome destabilization and acetylation may drive histones to proteasomal degradation. The designated nucleosome remodelers are engaged in nucleosome sliding, histone exchange, and/or disruption involving a displacement of histones from the damaged chromatin. After DNA damage is repaired, displaced histones may be re-deposited or re-positioned.

The “access-repair-restore” concept which describes how DNA lesions are rapidly and accurately repaired using a series of choreographed molecular processes, is well supported in the literature (210) (Fig. 2). To detect DNA damage efficiently and to provide access to the repair proteins at the damaged sites, the initial response to DNA damage depends on fast decompaction of surrounding chromatin at the damage site (211). Due to its high degree of compaction, heterochromatin shows resistance in the γ-H2AX phosphorylation foci formation, an initial step in the DDR process. Thus, the process of DDR in the heterochromatic regions differs from the euchromatic regions (212, 213). Additionally, inhibiting the function of histone deacetylases (HDACs), or lowering the levels of H1 histones increases DDR signaling and the extent of gamma H2AX spread from the damage site (214).

The correlation between limited repair factors access to chromatin and inefficient DDR process was revealed by an intriguing finding showing that nucleosome-free regions (NFRs) bound by transcription factors have higher mutation rates than the unbound NFRs (215, 216). Moreover, higher mutation rates are similarly correlated with higher nucleosome occupancy (215). However, even when transcribed genes are found in or close to heterochromatic zones, higher transcription rates typically correspond with lower occurrence of mutations on transcribed genes (217). These observations imply that gene expression reduces structural restrictions placed on DNA repair machinery by opening densely packed chromatin.

#### Role of chromatin in repairing DNA damage induced by exogenous agents

Compared to its role in preventing DNA damage and genomic instability induced by endogenous genotoxic agents, the role of chromatin structure in the repair of DNA damage induced by exogenous agents is better recognized. Hundreds of DNA-damaging events occur in cells on a daily basis whether in the post-mitotic or proliferating stage, and typically irrespective of the underlying sequence. DNA damage is caused by several exogenous factors with ultra-violet (UV) radiation being the most common cause of DNA damage. Exposure to UVC and UVB rays results in two types of nucleotide base dimerization lesions: cyclobutene pyrimidine dimers (CPDs) and 6-4 photoproducts (6-4PPs), if not removed these can cause mutations and genomic instability (198). Such bulky lesions are removed by NER, which is licensed for excision by conformational switching caused by a lesion blocking TFIIH translocation on DNA (218, 219). Exposure to ionizing radiations (X- and γ-rays) leads to even more deleterious DNA damage in the form of DSBs that besides interfering with DNA replication and transcription, can also cause loss of nucleotides and chromosomal rearrangements.

Experimental evidences show that NER works efficiently in naked DNA but the presence of nucleosomes and heterochromatin can hinder the accessibility of repair proteins to DNA (205, 220, 221). NER's efficiency depends on many factors and the lesion removal efficiency can differ significantly. For example, in non-transcribed DNA, global genome NER (GG-NER) of 6-4PPs is much faster than the GG-NER of UVC-induced CPDs (205, 220, 221). Chromatin may exert substantial influence on this lesion-specific repair, as evidence implies 6-4PPs predominantly form in internucleosomal regions, contrastingly CPDs are located equally in nucleosomal and internucleosomal DNA (222). Lesion removal in actively transcribed DNA occurs more rapidly than in non-transcribed DNA, likely because both GG-NER and transcription coupled NER remove these lesions, and chromatin is made more accessible by the transcription machinery (223).

Another exemplary source of damage comes from the DNA modifying agents, such as diethyl sulfate, ethyl methyl sulfate, and methyl methane sulfonate that add adducts to the DNA and are used for cancer therapies. These adducts are a barrier to DNA and RNA polymerases, thus impacting both replication as well as transcription. If not removed, adducts can lead to the formation of ssDNA stretches that are prone to damage, SSBs, and on occasion conversion of SSBs to DSBs in a replication-dependent manner.

DSBs, which can lead to chromosome instability and large-scale genetic alterations, can arise from damaging agents including IRs and genotoxic chemicals, or due to replication failure and/or replication stress. DSBs represent major hurdles to cells for survival, as it can directly lead to chromosome instability and large-scale genetic alterations. DSBs are mainly repaired by either HR (224) or NHEJ (201). HR is an error-proof repair pathway that preferentially uses a sister chromatid as a template to repair DNA damage, which is only available in the late S- or G2-phase of the cell cycle (225). By contrast, NHEJ occurs in all phases of the cell cycle, which fuses broken DNA ends together to repair the damage. Prior to NHEJ-associated ligation, limited DNA end-processing may cause loss of a few nucleotides, making NHEJ a more error-prone repair process than HR. Both DSB repair mechanisms begin with the detection of the breaks and subsequent DNA end processing, associated with extensive phosphorylation and ubiquitylation events that alter the chromatin and proteins surrounding the breaks (226). Histone H2AX is phosphorylated by phosphatidylinositol 3-(PI3) kinases such as ATM and ATR, adjacent to the DNA breaks leading to the recruitment of mediator factors, such as scaffold protein MDC1 and the signaling proteins BRCA1 and 53BP1/TP53BP1 (181, 227).

Chromatin structure also plays a major role in DSB repair and signaling. Chromatin compaction influences cell sensitivity to DSBs and DDR efficiency (228). Furthermore, DSBs in heterochromatin are repaired at a slower rate compared with the DSBs present in the euchromatin and require the ATM-dependent phosphorylation of heterochromatin protein KAP1/TRIM28 (229, 230). Moreover, chromatin relaxes upon DSB induction (150, 230, 231) and expands, and unwraps locally in an ATP-dependent fashion [88]. Thus the effectiveness of the DSB-associated DDR depends upon and modulates chromatin structure.

Experiments with localized UV-irradiation reveal local decompaction and a global chromatin relaxation response influenced by p53. UV damage is also reported to increase the unwinding of nucleosomes, shifting the equilibrium between winding and unwinding towards excess ‘DNA breathing’. This increases the time window for the repair of lesions in chromatin by facilitating repair factor access, lesion recognition, and binding that may further unwrap the DNA (232). Site-specific chromatin remodeling involves the crucial NER proteins, CSB/ERCC6 containing a SWI2/SNF2 ATPase domain that is indispensable for repair (233, 234, 235, 236). CSB remodel chromatin *in vitro* in an ATP-dependent fashion (237), and is required for NER factors recruitment to sites of transcription coupled NER. A photoactivatable GFP-tagged H2B experiments showed that chromatin experiences an ATP-dependent local expansion soon after the DNA damage (238). This regional expansion is independent of H2AX and ATM, the two early effectors of DDR, and correlates to a 30 to 40% drop in chromatin fiber density near DSBs (1, 239). Changes in histone PTMs, the mobility of chromatin-binding proteins, or nucleosome disruption can all impact on chromatin relaxation (240).

Chromatin rearrangement removes the barrier to repair machinery access as evident in a local temporary drop in density of core and linker histones at UVC damage sites. This is caused partly by the DNA damage binding protein 2 (DDB2)-encouraged ATP-dependent chromatin decompaction (241). UVA exposure reduces histone density during early chromatin decompaction as seen for a variety of linker histone variants and to a lower extent for core histones (H2A and H4) (242). In contrast to UVC and UVB, absorption by endogenous photosensitizers can cause oxidized bases (8-oxo-dG, thymidine-glycol), SSBs, DSBs, and other types of DNA lesions, (198, 243, 244).

Using chromatin immunoprecipitation (ChIP) techniques allows the effect of DSBs induced by endonucleases on histone dynamics to be directly examined, and data indicates a temporary dissociation of the core and linker histones from the chromatin at and around the damage site (245, 246). For X- and γ-rays, ionizing radiations and radiomimetic compounds that predominantly cause DSBs, all core histones except H2AZ undergo partial proteasome breakdown, and the resulting increased mobility facilitates homology search and repair (247). Besides the proteasome, Wss1 protease can also break down histones and during replication stress in yeast, Wss1 targets the non-specifically linked histones to the single-stranded DNA for degradation (248).

**Figure 3:**
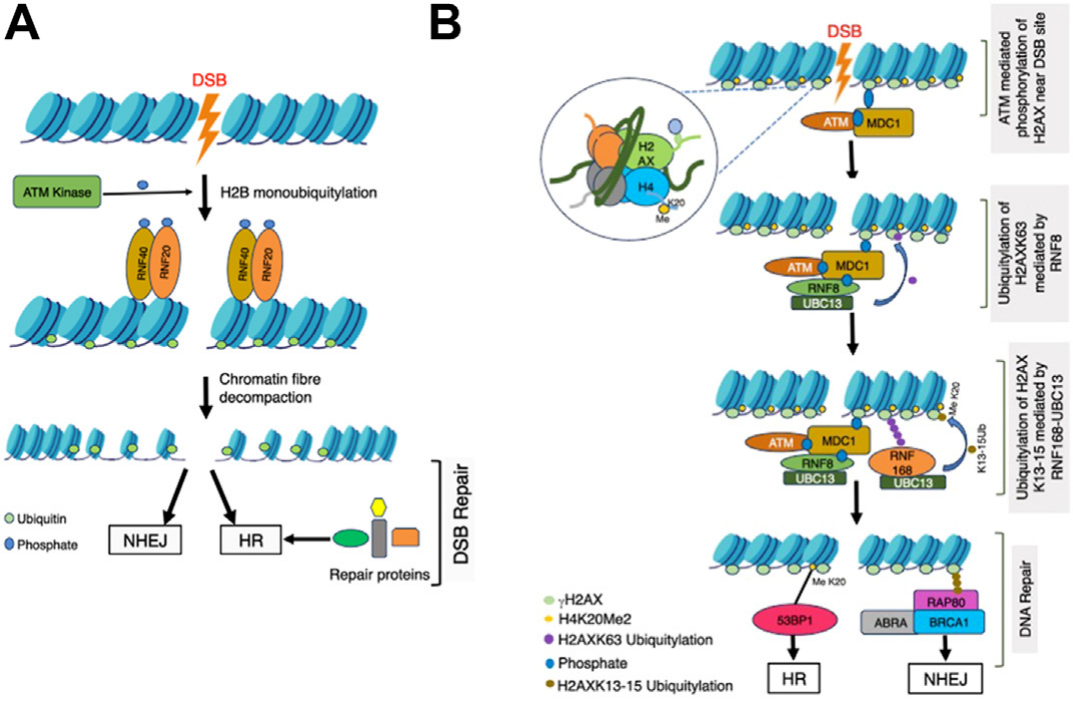
**Chromatin dynamics during DSB repair process.***A*, RNF20/40 mediated chromatin relaxation in DSB. ATM kinase, the primary transducer of double-stranded break (DSB) response, recruits and phosphorylates E3 ubiquitin ligase which is a heterodimer of the RING-finger proteins RNF20 and RNF40, at the DSB site. RNF20 and RNF40 further monoubiquitinylate at H2BK120 which is essential for timely accumulation of NHEJ and HR proteins at DSB sites and subsequent optimal repair *via* both pathways. *B*, RNF8/168-mediated ubiquitylation at DSB. In response to DSBs, ATM-mediated phosphorylation of γ-H2AX-bound with MDC1 generates binding sites for the RNF8 ubiquitin ligase to promote the formation of a ternary MDC1-RNF8-UBC13 complex at sites of DNA damage which further promote initial K63-linked polyubiquitylation of H2A-type histones. This in turn creates binding sites for the MIU domains of RNF168, allowing its recruitment which leads to ubiquitylation of K13-15 of H2A/H2AX at DSBs. Polyubiquitylation of the DSB-flanking chromatin mediated by RNF168 allows accumulation of genome caretaker proteins such as 53BP1 and the BRCA1. The relative dynamics with which these two components accumulate at break sites is significant in determining the choice of repair pathway the cell takes to guarantee genome stability.

Numerous histone PTMs control chromatin accessibility and histone dynamics. To regulate chromatin relaxation following DNA damage, core histone acetylation and ubiquitylation work in concert and a well-orchestrated fashion. H4 acetylation and H2B ubiquitylation, for instance, work collaboratively to enhance chromatin fiber decompaction (249) (Fig. 3). Following radiomimetic treatment, ring finger protein (RNF20-RNF40) ubiquitin ligase monoubiquitylates H2B on K120 in human cells (250) (Fig. 3A). Improper accumulation of DSB repair factors in cells deficient of RNF20 is prevented by substances that produce chromatin relaxation, illustrating how chromatin structure can able prediction and control of DNA damage responses (251). Uncertainly concerns whether H2B monoubiquitylation influences direct chromatin decompaction or this is mediated by chromatin remodeler sucrose nonfermenting 2 homologues (SNF2H) recruited by RNF20 as observed at heterochromatic DSBs (252). CUL4-dependent ubiquitylation of H2A, H3, and H4 upon detection of UV damage in human cells is hypothesized to decrease histone-DNA interactions, destabilize nucleosomes, and increase accessibility of repair components (253).

ADP-ribosylation is gaining attention as a PTM that controls damaged chromatin compaction in addition to acetylation and ubiquitylation. Through the activation of chromatin remodelers, ADP-ribosylation enhances chromatin relaxation at sites of UVA laser damage (254) and promotes histone removal at the DSBs (255). ADP-ribosylation on histone proteins increases immediately after oxidative damage (256). This suggests that histone ADP-ribosylation is a probable early mediator of chromatin relaxation by removing histones and recruiting chromatin remodelers. Notably, at damage sites, the poly (ADP-ribose) polymerases are the primary targets of ADP-ribosylation (257). This modification is then rapidly removed in minutes by the PARG glycohydrolase, and inhibitors are under investigation for the development of potential therapeutic agents for cancer therapy (258).

Among the important DNA damage-induced histone PTMs, γ-H2AX received the most attention initially. Upon DNA damage with a variety of agents, DNA damage-sensitive kinases quickly phosphorylate H2AX on an evolutionarily conserved carboxy-terminal serine over many megabases surrounding the lesion (259). The γ-H2AX then acts as a platform for the recruitment of damage signal-related proteins and setting off a cascade of histone ubiquitylation events, impacting DSB repair pathway choice (190, 258, 260, 261). Upon γ-H2AX formation, the RING-type ubiquitin ligase RNF8 (262) ubiquitinates K63 on H1 in the UBC13-dependent manner followed by the RNF168 mediated mono-ubiquitylation of H2A/H2AX on K13-K15 to facilitate the binding of NHEJ-promoting factor 53BP1 to the damage site (263) (Fig. 3B). RNF8/RNF168 lengthens ubiquitin chains on H2AK13/K15, thereby, facilitating the binding of HR promoting E3 ligase BRCA1-BARD1 complex *via* Rap80 subunit (264). Additionally, BRCA1-BARD1 helps in recruiting chromatin remodeler SMARCAD1 by catalyzing H2A ubiquitination on carboxy-terminal residues K127 to 129, displacing 53BP1 and encouraging end resection to facilitate DSB repair by HR (265). Apparently, histone H2A/H2AX is a key target for ubiquitylation in response to DSBs, with different residues both in the amino- and carboxy-termini acting as crucial factors in the choice of the DSB repair pathways. Damage-induced changes to histone H2B are less well understood; however, upon DSB induction in mammalian cells, SAGA (Spt-Ada-Gcn5 acetyltransferase) complex deubiquitinates and acetylates H2BK120, promoting DSB repair either by NHEJ or HR (266).

### The role of histone dynamics in restoring chromatin structure and function

Following DNA repair, multiple processes are put into action to restore chromatin organization. Histone chaperones play a crucial role in restoring chromatin architecture, as shown by ChIP analyses of histone proteins near the endonuclease cut sites in yeast and human cells. Anti-silencing function 1 (ASF1), chromatin assembly factor-1 (CAF-1), and histone regulator A (HIRA) co-ordinate to restore H3 occupancy around the DSB sites (267). Importantly, newly synthesized histones are deposited to restore the chromatin after repair as shown in human cells by transiently transfecting histones that were epitope-tagged (268) and by SNAP-tag technology for fluorescence tracking of novel histone dynamics (269). These methods found that the CAF-1 histone chaperone incorporates H3.1 histones *de novo* during the repair of UV lesions (269), whereas newly synthesized H3.3 histones are deposited by HIRA histone chaperon complex connected to UV damage detection (269). As shown in UVA microirradiated human cells, repair of chromatin structure also entails the re-establishing of histone H1 (242).

Besides the deposition of new histones, during chromatin repair, histone PTMs are maintained or dynamically regulated as a sequel to the DNA damage-response that is little researched despite extensive mapping of damage-induced histone PTMs. Multiple phosphatases and ubiquitin-specific proteases (USP) facilitate the prompt release of repair agents to aid in chromatin activation for transcription restart. After repair, the chromatin transcriptional activity must be reinstated as well as its structural integrity as exemplified by USP16 deubiquitinase, which eliminates ubiquitylation on H2AK11 (270). Histone chaperones also regulate transcription recovery upon DNA damage responses as HIRA and FACT histone chaperones encourage transcription resumption in human cells following UVC damage repair (271). H3.3 contributes to the activation of gene expression in both normal and pathological situations (272).

## DNA damage repair in heterochromatic regions

Under normal conditions, heterochromatin is compact and inaccessible to RNA polymerases. As discussed above, heterochromatin in most of the eukaryotic genome is represented as a repetitive sequence (140) consisting of tandem repeat “satellite” sequences and TEs (141). To maintain genome integrity and functional and structural regulation of these repetitive sequences, chromatin regulation of TEs is necessary (142). Although hetero-chromatinization of the repetitive sequences has evolved to safeguard the genome from instability, this on the other hand poses a challenge due to its restrictive nature.

**Figure 4:**
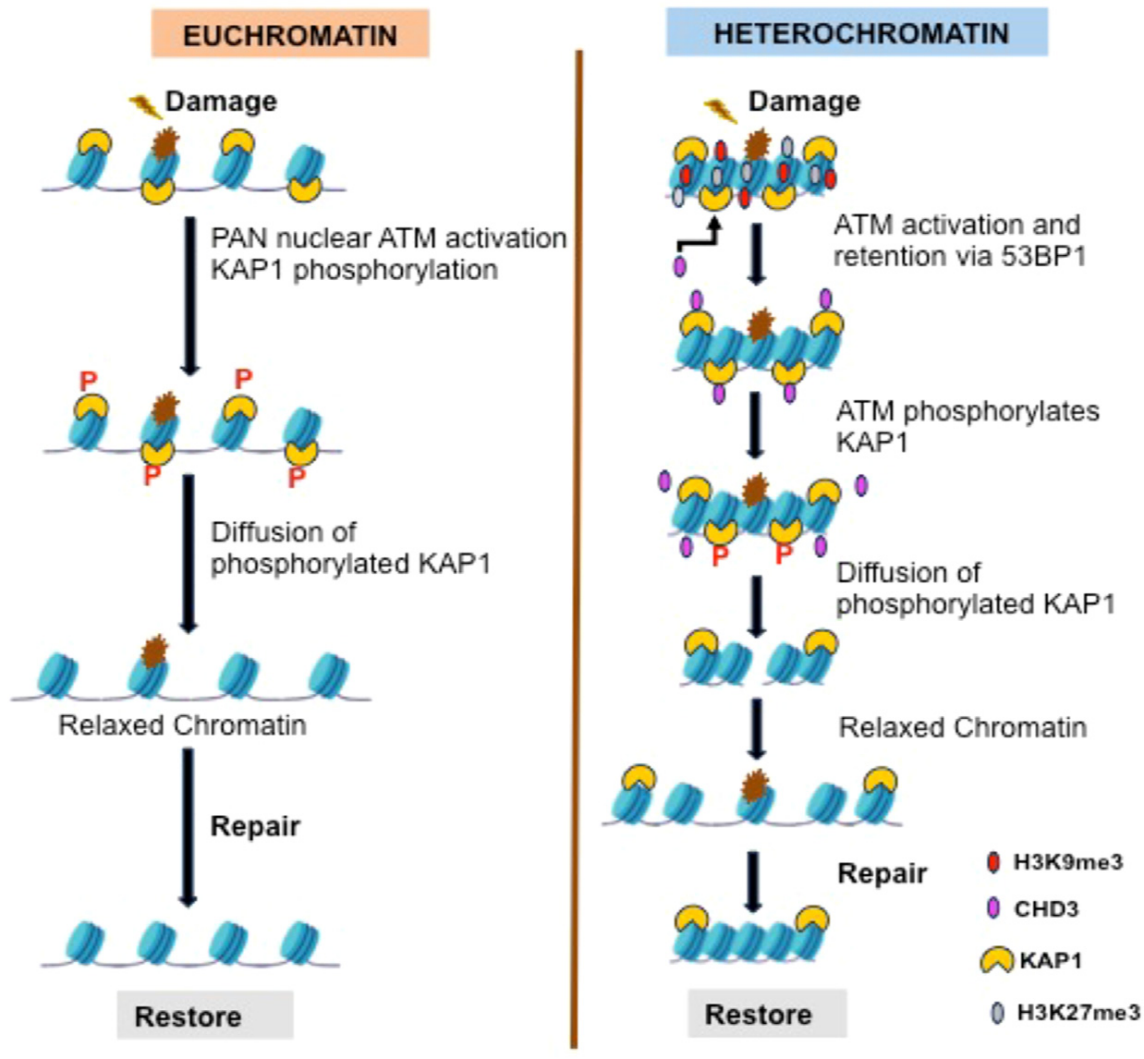
**DNA damage reaction in Heterochromatin and Euchromatin.** DNA DSBs in the Euchromatic region of the genome lead to the global activation of ATM kinase, which in turn leads to the diffused phosphorylation of KAP1. Phosphorylation of KAP1 and its release from chromatin promotes repair by increased access to the site of damage. When damage occurs in heterochromatin regions, ATM activity is maintained at the site where DSB is found, in a way that is dependent on 53BP1. This allows 53BP1 to maintain several factors on the site of damage and also allows for localized phosphorylation of local KAP1 and CHD3 dissociation resulting in chromatin relaxation.

The restrictive nature of heterochromatin mainly depends on repressive histone marks (H3K9me3 and H3K27me3), dense packing of nucleosomes, and the existence of other heterochromatin proteins such as HP1 and KAP-1 (230) (Fig. 4). During the DDR, DNA accessibility within heterochromatin may alter (273, 274, 275). The genomic DNA organization into chromatin fiber safeguards against break induction at basic nucleosomal level as well as at higher order chromatin structures. Also, chromatin in its decompaction state is more prone to damage caused by chemical agents like cisplatin (276). In *Drosophila*, compaction into heterochromatin state is favorable for survival when there is hydroxyurea (HU)-induced DNA damage (1). The repair process within the heterochromatin regions may be slower than the euchromatin regions and DSB repair within heterochromatic DSB occur after relocation of DSBs away from the repressive compartment (274). In *Drosophila,* the repair mechanism is similar for both euchromatic and heterochromatic domains (277). Yet in human cells, induced DSBs shift away from heterochromatin, suggesting a conserved segregation of HR-mediated repair that is away from the repetitive sequence rich domains (274, 278, 279).

In fact, studies suggest that DNA damage induces the folding of heterochromatin (280, 281). For example, in human fibroblasts and mouse NIH3T3 cells, in response to interferon-induced riboside, chromatin relaxation is induced by ATM-dependent phosphorylation of the KAP-1 and subsequent loss of this protein to heterochromatin (Fig. 4). Interestingly, chromatin remodeler chromodomain helicase DNA binding 3, (Chromodomain Helicase DNA Binding Protein 3) which acts in the compaction of chromatin and gene repression, is dissociated upon phosphorylation of KAP-1 (150, 274, 282) and after treatment with oxidative agent tert-butyl hydroperoxide (TBH). Yet, SWI/SNF chromatin remodeler is required to facilitate the invasion of Rad51-and Rad54-dependent strands during the recombinational repair of the mating type (MAT) locus with homothallic mating left (HML) (283).

## Genome instability in chromosomal fragile sites

Distinct from fundamental constrictions of the centromeres, the phrase “fragile site” as first used in 1969–70 describes the unique secondary constrictions in chromosomes (284). These fragile sites can also appear as gaps, failed chromatin compaction or chromatin cracks on metaphase chromosomes under certain replicative stress circumstances. Furthermore, most fragile locations are inherited within families, making them extremely conserved during chromosomal evolution. The fragile sites in the genome may arise from DNA's incapacity to fold compactly during metaphase and the appearance of the fragile spots may be influenced by elements like the DNA itself, histones, and non-histone proteins (285). Since chromatin structure can actively participate in DNA metabolic functions like transcription, replication, recombination, and repair, many, if not all, of these cellular functions are directly related to fragile spots causing instability (286, 287).

In the human genome, over a hundred fragile sites have been identified and these fragile sites are categorized as common fragile sites (CFSs) and rare fragile sites (RFSs) based on their frequency in the population and are further subdivided based on the agents used to identify them in cultured cells [Table 2]. About 90 CFSs and 30 RFSs that have been cytogenetically seen and recorded in earlier investigations are listed in the Human Genome Database (162). CFSs are induced by aphidicolin, 5-azacytidine, and bromodeoxyuridine (BrdU) and are prevalent in a significant section of population (288). The RFSs on the other hand are detected in the population at a maximum frequency of 5% (289) and are induced by folate deficiency/thymidylate stress, Distamycin A, and BrdU (290, 291).

**Table 2:** Classification of fragile sites

Rare fragile sites	Folate sensitive	FRA1M, FRA2A, FRA2B, FRA2K, FRA2L, FRA5G, FRA6A, FRA7A, FRA8A, FRA9A, FRA9B.FRA10A, FRA11A, FRA11B, FRA12A, FRA12D, FRA16A, FRA18C, FRA19B, FRA20A, FRA22A, FRAXA, FRAXE, FRAXF
	Distamycin-A inducible	FRA8E, FRA11I, FRA16E, FRA16B
	BrdU inducible	FRA17A, FRA10B
Common fragile sites	Aphidicolin inducible	FRA14B, FRA14C, FRA15A, FRA16C, FRA16D, FRA17B, FRA18A, FRA18B, FRA20B, FRA22B, FRAXB, FRAXC, FRAXD
	5-azacytidine inducible	FRA1H, FRA1J, FRA9F, FRA19A
	BrdU inducible	FRA4B, FRA5A, FRA5B, FRA6D, FRA9C, FRA10C, FRA13B

There are presently 25 aphidicolin-inducible CFSs that are molecularly mapped (162). They are all distinguished by extensive AT-rich DNA sections that range from hundreds of kilobases to megabases on a chromosome (292). They are often linked to hotspots of translocations, rearrangements, and deletions in cancer. Twenty-four of thirty known RFSs—referred to as folate-sensitive fragile sites (FSFS) appear during thymidylate stress, caused by the folate deficiency. Expanded repetitive DNA sequences have been discovered at every rare fragile site sequenced thus far, however, distinct repeat sequences have been discovered in each category. Thus far, ten FSFSs have been identified through sequence mapping to gene-specific expanded (CGG) repeats; the most well-known location being FRAXA, which is found at the fragile X messenger ribonucleoprotein 1 (*FMR1*) gene and results in fragile X syndrome (FXS). Moreover, minisatellite AT-rich repeat sequences have been linked to two of the distamycin A-inducible RFSs (293). Upon analyzing the DNA of a rare distamycin A-induced fragile site, FRA16B, an AT-rich repeat of 33 base pairs was discovered (ATATATTATATATTA TATCTAATAATATC/ATA) and this 33-bp repeat is amplified by up to 2000 copies, as opposed to 7 to 12 copies in the general population (294). Since CFSs are mechanistically and strongly associated with areas of chromosomal rearrangements in cancer, this category of fragile sites has received higher attention compared to the RFSs (295, 296, 297).

**Figure 5:**
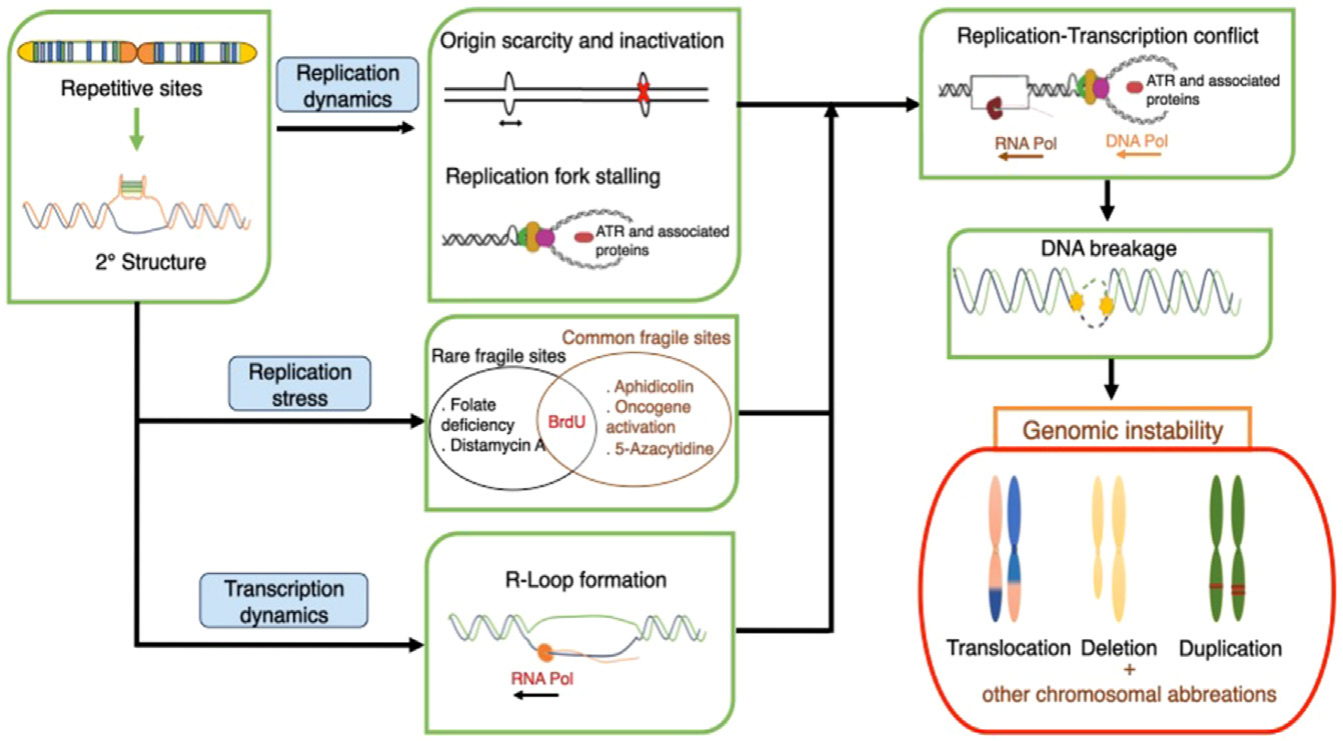
**Fragile site associated genomic instability.** An overview of the Chromosomal fragile sites and ultimate effects on genomic stability.

Emerging data points to multiple causes for CFS instability, including events that obstruct the replication process directly and the inherent features of fragile areas (Fig. 5). According to a structural examination of the CFS sequences, high flexibility sequences that show large variations in the twist angle between each pair of base pairs throughout the DNA sequence are abundant in FRA3B, FRA7H, and FRA7G (298, 299).

A selection of fragile sequences subjected to computational analysis revealed that CFSs frequently contain AT-rich islands (300). Due to their flexibility, these AT-rich sequences readily fold into secondary structures during the unwinding of the DNA double helix. Their tendency to adopt these structures may make them more susceptible to replication elongation disruption or fork stalling. The recapitulation of a CFS-like phenotype when FRA3B was integrated into a non-fragile locus is perhaps the most convincing evidence suggesting that fundamental properties of a CFS sequence are associated with breakage (301). Finding overlaps between AT- Di nucleotide-rich sequences (AT-DRSs) and recurring cancer breakpoints in CFSs provided direct evidence of the function of AT-DRSs in DNA instability *in vivo* (302, 303). The presence of long tracts of dA:dT homopolymers in the early replicating fragile sites (ERFSs), CFSs, and ribosomal DNA pose a challenge to the replication machinery and are ideal places for the replication fork to stall and collapse under the conditions of replication stress (304). Because the long dA-rich strand is not protected by replication protein A (RPA) when DNA unwinds at the replication fork, poly(dA:dT) tracts are more likely to create non-B DNA structures which may stop DNA synthesis. These new discoveries highlight the significance of various sequences that can generate non-B DNA structures in the CFSs (Fig. 5).

In comparison to the open/active regions of the genome, CFSs are typically hypoacetylated, suggesting their compact organization (305). These regions show resistance to micrococcal nuclease (MNase) activity, which further supports their compactness (306). Recently, FSFS FRA2L was shown as the cause for kink or bending of chromosome 2 under folate stress conditions due to the presence of CCG trinucleotide repeats in this region (307). Therefore, in addition to the epigenetic effects, the stability of fragile sites may be impacted by the development of unique DNA structures. Mechanistically, these secondary structures disrupted the lengthening of DNA replication both *in vivo* and *in vitro*, which is probably how they cause fragility.

The preservation of CFS integrity throughout replication is linked to a wide range of cellular processes, many of which are also involved in processing the structural barriers that arise along these regions during replication. Stabilizing factors that function during the S-phase and factors that process replication intermediates after the S-phase make up the known mechanisms. Proteins that can resolve DNA secondary structures or stabilize stalled forks, as well as specialized DNA polymerases involved in synthesis at complicated DNA sequences, are important participants during S-phase. ATR kinase senses and responds to DNA replication stress by phosphorylating restart nuclease EXO5 in complex with the BLM helicase, directing replication fork restart to maintain genome stability (308). The resolution of other DNA secondary structures depends on the helicase and exonuclease activities of the Werner protein (WRN), which belongs to the RecQ family of exonucleases (203, 309, 310). Even in normal cells, gaps and constrictions on metaphase chromosomes at CFSs are linked to WRN deficiency (311).

The replicative helicase causes torsional stress on DNA during replication. This is relieved by topoisomerase l (Topo l) by cleaving one strand of duplex DNA, unraveling the strand, and relegating the cleavage site (312). The carboxy-terminal binding protein (CtBP)-interacting protein (CtIP) is also linked to preserving the stability of repetitive sequences and CFSs. In order to assist the Mre11/Rad50/Nbs1 (MRN) complex in mediating DSB resection, ATR activation, and homologous recombination repair, CtIP is recruited to DNA damage sites (203, 310, 313, 314). Fanconi anemia (FA) proteins are also reported important for controlling CFS stability and responding to replication stress (315). The replication machinery is obstructed overall by secondary structure creation, which results in fork arrest and DSBs. To preserve the stability of CFS, many cellular proteins must specialize in overcoming such replication obstacles.

Given their capacity to replicate non-B DNA structures and their function in resuming stalled replication forks, specialized DNA polymerases represent an important intra-S-phase maintenance arm. These polymerases reduce DNA damage transmission to daughter cells (316). According to multiple studies, the specialized polymerases eta (Pol η), kappa (Pol ĸ), and zeta (Pol ζ) which may avoid structured DNA, temporarily substitute for the stalled replicative DNA polymerase delta (Pol δ) (317, 318, 319, 320). The Pol δ stalling mechanism in CFSs may be that Pol δ dissociates at sequence segments containing mononucleotides [A]n and dinucleotides [TA]n (317). Following treatment with aphidicolin, Pol ƞ is recruited to genomic areas that map at hot sites for DNA breaking inside FRA7H and FRA16D (318). Pol ƞ and Pol κ are more effective at replicating FRA16D and FRA3B-derived AT-DRSs that are expected to create non-B DNA secondary structures than the replicative Pol δ (317, 318). Replication forks that have stalled can be saved by these specialized DNA polymerases, mainly through their involvement in S-phase checkpoint activation (316). Repeat sequences and secondary DNA structures act in mechanisms leading to CFS instability, as supported by the important roles for specific proteins and polymerases that are capable of overcoming replication barriers to maintain CFS stability (Fig. 5). However, these processes induce some instability as the replication through CFSs by Pol ƞ appears to result in genetic variations found in the human population at these sites (321).

## Conclusion

Consideration of the intricate relationship between chromatin structure and genome stability described here uncovers multiple specific ways that chromatin dynamics guide transcription and DNA repair processes and safeguard genomic integrity. Chromatin is not only a key structural scaffold: it is a dynamic entity crucial for regulating most DNA-dependent functions and accurately preserving genetic information. Genomic instability arises from various sources, including DSBs and impaired recombination events and even replication, particularly in the repetitive DNA regions prone to replication error as well as to transcriptional and transpositional activities. Heterochromatinization of repetitive sequences restricts their unnecessary transcription and mobility, thereby mitigating the risk of genome destabilization. However, the transcriptional activity of repetitive elements introduces complexity, questioning whether all repeats may pose equal risks to genomic integrity. Moreover, chromatin regulation involves a sophisticated interplay of chromatin remodelers, histone chaperones, and histone-modifying enzymes that sculpt chromatin architecture to regulate transcription and establish a landscape conducive to repair processes. Dynamic chromatin compaction, balancing accessibility for repair with structural constraints to prevent genomic instability, underscores its critical role in DNA repair and genome stability mechanisms that we need to better define for predictive biology.

## Future perspective

It is timely to systematically build the comprehensive database needed to define pairwise relationships that address the emerging grand challenge of connecting chromatin organizational patterns to functional outcomes for transcription, DNA repair, and genomic integrity. A detailed mechanistic knowledge of variables and alterations for chromatin structure and its adaptive response to both endogenous and external damage remains an elusive but key goal. Importantly, the tools now exist to define the chromatin architecture nexus regulating transcription and DNA damage repair. For example, recent discoveries imply that chromatin shape affects pathway repair decisions for HR and NHEJ. Open chromatin configurations generally facilitate HR-mediated repair efficiency, whereas compact heterochromatin poses challenges due to restricted accessibility. Building a database defining how patterns of chromatin dynamics inform strategies to enhance repair mechanisms and mitigate genomic instability is technically feasible and likely to be powerful and important. Furthermore, current reporter assays for DNA repair are limited in that they only report on open actively transcribed chromatin. As we find that chromatin conformation is important in determining some of the repair outcomes, we recommend incorporating comparative studies that introduce defined damaged sites such as DSBs in transcribing and non-transcribing chromatin regions to unveil the effects of chromatin where feasible.

The dramatic impact of chromatin structural perturbations on the proper orchestration of repair, transcription, and replication dynamics underscores the importance of relating their specific pairwise and nuanced relationships to genome stability and instability. Furthermore, delineating the choreography of transcription and repair responses resulting from chromatin structure in different cells and tissues will distinguish pleiotropic and context-specific relationships due to distinct chromatin modifications and protein complexes. We noted that perturbed chromatin states can lead to deregulated DNA-templated activities, potentially culminating in genomic failures such as chromosomal rearrangements and mutations. Defining how chromatin disruptions influence specific cellular responses for replicative, transcriptional, and environmental stresses will provide the key knowledge base for predictive insights into the maintenance of genomic stability. Currently, fragile site expression at specific loci exemplifies the complex interplay of chromatin structure and cellular vulnerability. Elucidating the molecular underpinnings behind fragile site expression and the differential cell responses to different stress situations is an exemplary window into more predictive cell biology. Growing this information is likely to enhance actionable knowledge of genomic instability mechanisms and guide directed strategies to mitigate their adverse consequences in disease including both cancer and neurodegenerative diseases.

## Data availability

All supporting data are provided within the manuscript, supplementary data, and supplementary tables.

## Conflict of interest

The authors declare that they have no conflicts of interest with the contents of this article.

## References

[1] Nair N., Shoaib M., Sorensen C.S. (2017). Chromatin dynamics in genome stability: roles in suppressing endogenous DNA damage and facilitating DNA repair. Int. J. Mol. Sci..

[2] Touchon M., Rocha E.P. (2016). Coevolution of the organization and structure of prokaryotic genomes. Cold Spring Harb Perspect. Biol..

[3] diCenzo G.C., Finan T.M. (2017). The divided bacterial genome: structure, function, and evolution. Microbiol. Mol. Biol. Rev..

[4] Hammel M., Amlanjyoti D., Reyes F.E., Chen J.H., Parpana R., Tang H.Y. (2016). HU multimerization shift controls nucleoid compaction. Sci. Adv..

[5] Sinden R.R., Pettijohn D.E. (1981). Chromosomes in living Escherichia coli cells are segregated into domains of supercoiling. Proc. Natl. Acad. Sci. U. S. A..

[6] Dorman C.J., Dorman M.J. (2016). DNA supercoiling is a fundamental regulatory principle in the control of bacterial gene expression. Biophys. Rev..

[7] Das C., Bhattacharya A., Adhikari S., Mondal A., Mondal P., Adhikary S. (2024). A prismatic view of the epigenetic-metabolic regulatory axis in breast cancer therapy resistance. Oncogene.

[8] Misri S., Pandita S., Pandita T.K. (2009). Detecting ATM-dependent chromatin modification in DNA damage and heat shock response. Methods Mol. Biol..

[9] Bickmore W.A., van Steensel B. (2013). Genome architecture: domain organization of interphase chromosomes. Cell.

[10] Bonev B., Cavalli G. (2016). Organization and function of the 3D genome. Nat. Rev. Genet..

[11] Banday S., Pandita R.K., Mushtaq A., Bacolla A., Mir U.S., Singh D.K. (2021). Autism-associated vigilin depletion impairs DNA damage repair. Mol. Cell Biol..

[12] Bacolla A., Sengupta S., Ye Z., Yang C., Mitra J., De-Paula R.B. (2021). Heritable pattern of oxidized DNA base repair coincides with pre-targeting of repair complexes to open chromatin. Nucleic Acids Res..

[13] Horton C.A., Alexandari A.M., Hayes M.G.B., Marklund E., Schaepe J.M., Aditham A.K. (2023). Short tandem repeats bind transcription factors to tune eukaryotic gene expression. Science.

[14] Bacolla A., Ye Z., Ahmed Z., Tainer J.A. (2019). Cancer mutational burden is shaped by G4 DNA, replication stress and mitochondrial dysfunction. Prog. Biophys. Mol. Biol..

[15] Abramson J., Adler J., Dunger J., Evans R., Green T., Pritzel A. (2024). Accurate structure prediction of biomolecular interactions with AlphaFold 3. Nature.

[16] Hajjoul H., Mathon J., Ranchon H., Goiffon I., Mozziconacci J., Albert B. (2013). High-throughput chromatin motion tracking in living yeast reveals the flexibility of the fiber throughout the genome. Genome Res..

[17] Gu B., Swigut T., Spencley A., Bauer M.R., Chung M., Meyer T. (2018). Transcription-coupled changes in nuclear mobility of mammalian cis-regulatory elements. Science.

[18] Ma H., Tu L.C., Naseri A., Huisman M., Zhang S., Grunwald D. (2016). Multiplexed labeling of genomic loci with dCas9 and engineered sgRNAs using CRISPRainbow. Nat. Biotechnol..

[19] Horsfield J. (2025). Cohesin and CTCF emerge as building blocks of 3D genome structure. Nat. Rev. Genet..

[20] Ertl H. (2024). The regulatory landscape of chromatin accessibility. Nat. Rev. Genet..

[21] Yu Q., Liu X., Fang J., Wu H., Guo C., Zhang W. (2023). Dynamics and regulation of mitotic chromatin accessibility bookmarking at single-cell resolution. Sci. Adv..

[22] Coux R.X., Owens N.D.L., Navarro P. (2020). Chromatin accessibility and transcription factor binding through the perspective of mitosis. Transcription.

[23] Zhou M.M., Cole P.A. (2025). Targeting lysine acetylation readers and writers. Nat. Rev. Drug Discov..

[24] Paliwal S., Dey P., Tambat S., Shinohara A., Mehta G. (2024). Role of ATP-dependent chromatin remodelers in meiosis. Trends Genet..

[25] Alvarez-Gonzalez L., Ruiz-Herrera A. (2025). Evolution of 3D chromatin folding. Annu. Rev. Anim. Biosci..

[26] Gui Y., Li L., Wang J. (2024). [Anomalies of ATP-dependent chromatin remodeling complexes and human neurodevelopmental genetic disorders]. Zhonghua Yi Xue Yi Chuan Xue Za Zhi.

[27] Farnung L. (2025). Chromatin transcription elongation - a structural perspective. J. Mol. Biol..

[28] Clyde D. (2021). GETting at single-cell chromatin dynamics. Nat. Rev. Genet..

[29] Koch L. (2020). Tracing chromatin architecture. Nat. Rev. Genet..

[30] Müller S., Almouzni G. (2017). Chromatin dynamics during the cell cycle at centromeres. Nat. Rev. Genet..

[31] Pandita T.K., Richardson C. (2009). Chromatin remodeling finds its place in the DNA double-strand break response. Nucleic Acids Res..

[32] Sokolova V., Sarkar S., Tan D. (2023). Histone variants and chromatin structure, update of advances. Comput. Struct. Biotechnol. J..

[33] Luger K., Dechassa M.L., Tremethick D.J. (2012). New insights into nucleosome and chromatin structure: an ordered state or a disordered affair?. Nat. Rev. Mol. Cell Biol..

[34] Tremethick D.J. (2007). Higher-order structures of chromatin: the elusive 30 nm fiber. Cell.

[35] Hansen J.C. (2002). Conformational dynamics of the chromatin fiber in solution: determinants, mechanisms, and functions. Annu. Rev. Biophys. Biomol. Struct..

[36] Maeshima K., Imai R., Tamura S., Nozaki T. (2014). Chromatin as dynamic 10-nm fibers. Chromosoma.

[37] Woodcock C.L., Skoultchi A.I., Fan Y. (2006). Role of linker histone in chromatin structure and function: H1 stoichiometry and nucleosome repeat length. Chromosome Res..

[38] Maeshima K., Matsuda T., Shindo Y., Imamura H., Tamura S., Imai R. (2018). A transient rise in free Mg(2+) ions released from ATP-Mg hydrolysis contributes to mitotic chromosome condensation. Curr. Biol..

[39] Machida S., Takizawa Y., Ishimaru M., Sugita Y., Sekine S., Nakayama J.I. (2018). Structural basis of heterochromatin formation by human HP1. Mol. Cell..

[40] Sanulli S., Trnka M.J., Dharmarajan V., Tibble R.W., Pascal B.D., Burlingame A.L. (2019). HP1 reshapes nucleosome core to promote phase separation of heterochromatin. Nature.

[41] Mol C.D., Izumi T., Mitra S., Tainer J.A. (2000). DNA-bound structures and mutants reveal abasic DNA binding by APE1 and DNA repair coordination [corrected]. Nature.

[42] Tsutakawa S.E., Thompson M.J., Arvai A.S., Neil A.J., Shaw S.J., Algasaier S.I. (2017). Phosphate steering by Flap Endonuclease 1 promotes 5'-flap specificity and incision to prevent genome instability. Nat. Commun..

[43] Tsutakawa S.E., Sarker A.H., Ng C., Arvai A.S., Shin D.S., Shih B. (2020). Human XPG nuclease structure, assembly, and activities with insights for neurodegeneration and cancer from pathogenic mutations. Proc. Natl. Acad. Sci. U. S. A..

[44] Tubbs J.L., Latypov V., Kanugula S., Butt A., Melikishvili M., Kraehenbuehl R. (2009). Flipping of alkylated DNA damage bridges base and nucleotide excision repair. Nature.

[45] Maeshima K., Rogge R., Tamura S., Joti Y., Hikima T., Szerlong H. (2016). Nucleosomal arrays self-assemble into supramolecular globular structures lacking 30-nm fibers. EMBO J..

[46] Pepenella S., Murphy K.J., Hayes J.J. (2014). A distinct switch in interactions of the histone H4 tail domain upon salt-dependent folding of nucleosome arrays. J. Biol. Chem..

[47] Allis C.D., Jenuwein T. (2016). The molecular hallmarks of epigenetic control. Nat. Rev. Genet..

[48] Maeshima K., Iida S., Shimazoe M.A., Tamura S., Ide S. (2024). Is euchromatin really open in the cell?. Trends Cell Biol..

[49] Hildebrand E.M., Dekker J. (2020). Mechanisms and functions of chromosome compartmentalization. Trends Biochem. Sci..

[50] Radman-Livaja M., Rando O.J. (2010). Nucleosome positioning: how is it established, and why does it matter?. Dev. Biol..

[51] Ito S., Das N.D., Umehara T., Koseki H. (2022). Factors and mechanisms that influence chromatin-mediated enhancer-promoter interactions and transcriptional regulation. Cancers (Basel).

[52] Trojer P., Reinberg D. (2007). Facultative heterochromatin: is there a distinctive molecular signature?. Mol. Cell.

[53] Khorasanizadeh S. (2004). The nucleosome: from genomic organization to genomic regulation. Cell.

[54] Grewal S.I.S. (2023). The molecular basis of heterochromatin assembly and epigenetic inheritance. Mol. Cell.

[55] Bizhanova A., Kaufman P.D. (2021). Close to the edge: heterochromatin at the nucleolar and nuclear peripheries. Biochim. Biophys. Acta. Gene. Regul. Mech..

[56] Agbleke A.A., Amitai A., Buenrostro J.D., Chakrabarti A., Chu L., Hansen A.S. (2020). Advances in chromatin and chromosome research: perspectives from multiple fields. Mol. Cell.

[57] Janssen A., Colmenares S.U., Karpen G.H. (2018). Heterochromatin: guardian of the genome. Annu. Rev. Cell Dev. Biol..

[58] Zenk F., Zhan Y., Kos P., Loser E., Atinbayeva N., Schachtle M. (2021). HP1 drives de novo 3D genome reorganization in early Drosophila embryos. Nature.

[59] Strom A.R., Emelyanov A.V., Mir M., Fyodorov D.V., Darzacq X., Karpen G.H. (2017). Phase separation drives heterochromatin domain formation. Nature.

[60] Larson A.G., Elnatan D., Keenen M.M., Trnka M.J., Johnston J.B., Burlingame A.L. (2017). Liquid droplet formation by HP1alpha suggests a role for phase separation in heterochromatin. Nature.

[61] Boettiger A.N., Bintu B., Moffitt J.R., Wang S., Beliveau B.J., Fudenberg G. (2016). Super-resolution imaging reveals distinct chromatin folding for different epigenetic states. Nature.

[62] Cappa M., Bertini E., Di Capua M., Rimoldi M., Uziel G. (1990). A new therapeutic approach for X-linked adrenoleukodystrophy. Eur. J. Pediatr..

[63] Maeshima K., Ide S., Babokhov M. (2019). Dynamic chromatin organization without the 30-nm fiber. Curr. Opin. Cell Biol..

[64] Lieberman-Aiden E., van Berkum N.L., Williams L., Imakaev M., Ragoczy T., Telling A. (2009). Comprehensive mapping of long-range interactions reveals folding principles of the human genome. Science.

[65] Nora E.P., Lajoie B.R., Schulz E.G., Giorgetti L., Okamoto I., Servant N. (2012). Spatial partitioning of the regulatory landscape of the X-inactivation centre. Nature.

[66] Sexton T., Yaffe E., Kenigsberg E., Bantignies F., Leblanc B., Hoichman M. (2012). Three-dimensional folding and functional organization principles of the Drosophila genome. Cell.

[67] Nozaki T., Imai R., Tanbo M., Nagashima R., Tamura S., Tani T. (2017). Dynamic organization of chromatin domains revealed by super-resolution live-cell imaging. Mol. Cell.

[68] Xie L., Liu Z. (2021). Single-cell imaging of genome organization and dynamics. Mol. Syst. Biol..

[69] Ma H., Tu L.C., Chung Y.C., Naseri A., Grunwald D., Zhang S. (2019). Cell cycle- and genomic distance-dependent dynamics of a discrete chromosomal region. J. Cell Biol..

[70] Chen B., Gilbert L.A., Cimini B.A., Schnitzbauer J., Zhang W., Li G.W. (2013). Dynamic imaging of genomic loci in living human cells by an optimized CRISPR/Cas system. Cell.

[71] Ma H., Naseri A., Reyes-Gutierrez P., Wolfe S.A., Zhang S., Pederson T. (2015). Multicolor CRISPR labeling of chromosomal loci in human cells. Proc. Natl. Acad. Sci. U. S. A..

[72] Ohno M., Ando T., Priest D.G., Kumar V., Yoshida Y., Taniguchi Y. (2019). Sub-nucleosomal genome structure reveals distinct nucleosome folding motifs. Cell.

[73] Risca V.I., Denny S.K., Straight A.F., Greenleaf W.J. (2017). Variable chromatin structure revealed by in situ spatially correlated DNA cleavage mapping. Nature.

[74] Risca V.I. (2019). Nucleosome orientation map finds two new chromatin folding motifs. Cell.

[75] Kamat K., Lao Z., Qi Y., Wang Y., Ma J., Zhang B. (2023). Compartmentalization with nuclear landmarks yields random, yet precise, genome organization. Biophys. J..

[76] Redding S. (2021). Dynamic asymmetry and why chromatin defies simple physical definitions. Curr. Opin. Cell Biol..

[77] Newar K., Abdulla A.Z., Salari H., Fanchon E., Jost D. (2022). Dynamical modeling of the H3K27 epigenetic landscape in mouse embryonic stem cells. PLoS Comput. Biol..

[78] Johnstone C.P., Wang N.B., Sevier S.A., Galloway K.E. (2020). Understanding and engineering chromatin as a dynamical system across length and timescales. Cell Syst..

[79] De-Paula R.B., Bacolla A., Syed A., Tainer J.A. (2024). Enriched G4 forming repeats in the human genome are associated with robust well-coordinated transcription and reduced cancer transcriptome variation. J. Biol. Chem..

[80] Dudka D., Lampson M.A. (2022). Centromere drive: model systems and experimental progress. Chromosome Res..

[81] Talbert P.B., Henikoff S. (2020). What makes a centromere?. Exp. Cell Res..

[82] Talbert P., Henikoff S. (2022). Centromere drive: chromatin conflict in meiosis. Curr. Opin. Genet. Dev..

[83] Thakur J., Henikoff S. (2016). CENPT bridges adjacent CENPA nucleosomes on young human alpha-satellite dimers. Genome Res..

[84] Kasinathan S., Henikoff S. (2018). Non-B-Form DNA is enriched at centromeres. Mol. Biol. Evol..

[85] Nurk S., Koren S., Rhie A., Rautiainen M., Bzikadze A.V., Mikheenko A. (2022). The complete sequence of a human genome. Science.

[86] Henikoff J.G., Thakur J., Kasinathan S., Henikoff S. (2015). A unique chromatin complex occupies young alpha-satellite arrays of human centromeres. Sci. Adv..

[87] Thakur J., Henikoff S. (2018). Unexpected conformational variations of the human centromeric chromatin complex. Genes. Dev..

[88] Schalch T., Steiner F.A. (2017). Structure of centromere chromatin: from nucleosome to chromosomal architecture. Chromosoma.

[89] Bergmann J.H., Jakubsche J.N., Martins N.M., Kagansky A., Nakano M., Kimura H. (2012). Epigenetic engineering: histone H3K9 acetylation is compatible with kinetochore structure and function. J. Cell Sci..

[90] Sadeghi L., Siggens L., Svensson J.P., Ekwall K. (2014). Centromeric histone H2B monoubiquitination promotes noncoding transcription and chromatin integrity. Nat. Struct. Mol. Biol..

[91] Ribeiro S.A., Vagnarelli P., Dong Y., Hori T., McEwen B.F., Fukagawa T. (2010). A super-resolution map of the vertebrate kinetochore. Proc. Natl. Acad. Sci. U. S. A..

[92] Nakano M., Cardinale S., Noskov V.N., Gassmann R., Vagnarelli P., Kandels-Lewis S. (2008). Inactivation of a human kinetochore by specific targeting of chromatin modifiers. Dev. Cell.

[93] Ohzeki J., Shono N., Otake K., Martins N.M., Kugou K., Kimura H. (2016). KAT7/HBO1/MYST2 regulates CENP-A chromatin assembly by antagonizing Suv39h1-mediated centromere inactivation. Dev. Cell.

[94] Tachiwana H., Kagawa W., Shiga T., Osakabe A., Miya Y., Saito K. (2011). Crystal structure of the human centromeric nucleosome containing CENP-A. Nature.

[95] Roulland Y., Ouararhni K., Naidenov M., Ramos L., Shuaib M., Syed S.H. (2016). The flexible ends of CENP-A nucleosome are required for mitotic fidelity. Mol. Cell.

[96] Yatskevich S., Muir K.W., Bellini D., Zhang Z., Yang J., Tischer T. (2022). Structure of the human inner kinetochore bound to a centromeric CENP-A nucleosome. Science.

[97] Yan K., Yang J., Zhang Z., McLaughlin S.H., Chang L., Fasci D. (2019). Structure of the inner kinetochore CCAN complex assembled onto a centromeric nucleosome. Nature.

[98] Black B.E., Foltz D.R., Chakravarthy S., Luger K., Woods V.L., Cleveland D.W. (2004). Structural determinants for generating centromeric chromatin. Nature.

[99] Black B.E., Jansen L.E., Maddox P.S., Foltz D.R., Desai A.B., Shah J.V. (2007). Centromere identity maintained by nucleosomes assembled with histone H3 containing the CENP-A targeting domain. Mol. Cell.

[100] Fachinetti D., Han J.S., McMahon M.A., Ly P., Abdullah A., Wong A.J. (2015). DNA sequence-specific binding of CENP-B enhances the fidelity of human centromere function. Dev. Cell.

[101] Bai Y., Zhou Z., Feng H., Zhou B.R. (2011). Recognition of centromeric histone variant CenH3s by their chaperones: structurally conserved or not. Cell Cycle.

[102] Hu H., Liu Y., Wang M., Fang J., Huang H., Yang N. (2011). Structure of a CENP-A-histone H4 heterodimer in complex with chaperone HJURP. Genes. Dev..

[103] Nagpal H., Ali-Ahmad A., Hirano Y., Cai W., Halic M., Fukagawa T. (2023). CENP-A and CENP-B collaborate to create an open centromeric chromatin state. Nat. Commun..

[104] Chinnam N.B., Syed A., Hura G.L., Hammel M., Tainer J.A., Tsutakawa S.E. (2023). Combining small angle X-ray scattering (SAXS) with protein structure predictions to characterize conformations in solution. Methods Enzymol..

[105] Brosey C.A., Link T.M., Shen R., Moiani D., Burnett K., Hura G.L. (2024). Chemical screening by time-resolved X-ray scattering to discover allosteric probes. Nat. Chem. Biol..

[106] Palm W., de Lange T. (2008). How shelterin protects mammalian telomeres. Annu. Rev. Genet..

[107] de Lange T. (2005). Shelterin: the protein complex that shapes and safeguards human telomeres. Genes. Dev..

[108] Chen Y., Yang Y., van Overbeek M., Donigian J.R., Baciu P., de Lange T. (2008). A shared docking motif in TRF1 and TRF2 used for differential recruitment of telomeric proteins. Science.

[109] Fojtova M., Fajkus J. (2014). Epigenetic regulation of telomere maintenance. Cytogenet. Genome Res..

[110] Schoeftner S., Blasco M.A. (2009). A 'higher order' of telomere regulation: telomere heterochromatin and telomeric RNAs. EMBO J..

[111] Michishita E., McCord R.A., Berber E., Kioi M., Padilla-Nash H., Damian M. (2008). SIRT6 is a histone H3 lysine 9 deacetylase that modulates telomeric chromatin. Nature.

[112] Michishita E., McCord R.A., Boxer L.D., Barber M.F., Hong T., Gozani O. (2009). Cell cycle-dependent deacetylation of telomeric histone H3 lysine K56 by human SIRT6. Cell Cycle.

[113] Mostoslavsky R., Chua K.F., Lombard D.B., Pang W.W., Fischer M.R., Gellon L. (2006). Genomic instability and aging-like phenotype in the absence of mammalian SIRT6. Cell.

[114] Lachner M., O'Carroll D., Rea S., Mechtler K., Jenuwein T. (2001). Methylation of histone H3 lysine 9 creates a binding site for HP1 proteins. Nature.

[115] Jones B., Su H., Bhat A., Lei H., Bajko J., Hevi S. (2008). The histone H3K79 methyltransferase Dot1L is essential for mammalian development and heterochromatin structure. PLoS Genet..

[116] Schultz D.C., Ayyanathan K., Negorev D., Maul G.G., Rauscher F.J. (2002). SETDB1: a novel KAP-1-associated histone H3, lysine 9-specific methyltransferase that contributes to HP1-mediated silencing of euchromatic genes by KRAB zinc-finger proteins. Genes. Dev..

[117] Rosenfeld J.A., Wang Z., Schones D.E., Zhao K., DeSalle R., Zhang M.Q. (2009). Determination of enriched histone modifications in non-genic portions of the human genome. BMC Genomics.

[118] Benetti R., Garcia-Cao M., Blasco M.A. (2007). Telomere length regulates the epigenetic status of mammalian telomeres and subtelomeres. Nat. Genet..

[119] Sun Z.W., Allis C.D. (2002). Ubiquitination of histone H2B regulates H3 methylation and gene silencing in yeast. Nature.

[120] Rhie B.H., Song Y.H., Ryu H.Y., Ahn S.H. (2013). Cellular aging is associated with increased ubiquitylation of histone H2B in yeast telomeric heterochromatin. Biochem. Biophys. Res. Commun..

[121] Zhou L., Holt M.T., Ohashi N., Zhao A., Muller M.M., Wang B. (2016). Evidence that ubiquitylated H2B corrals hDot1L on the nucleosomal surface to induce H3K79 methylation. Nat. Commun..

[122] Galan A., Garcia-Oliver E., Nuno-Cabanes C., Rubinstein L., Kupiec M., Rodriguez-Navarro S. (2018). The evolutionarily conserved factor Sus1/ENY2 plays a role in telomere length maintenance. Curr. Genet..

[123] Tommerup H., Dousmanis A., de Lange T. (1994). Unusual chromatin in human telomeres. Mol. Cell Biol..

[124] Lejnine S., Makarov V.L., Langmore J.P. (1995). Conserved nucleoprotein structure at the ends of vertebrate and invertebrate chromosomes. Proc. Natl. Acad. Sci. U. S. A..

[125] Hubner B., von Otter E., Ahsan B., Wee M.L., Henriksson S., Ludwig A. (2022). Ultrastructure and nuclear architecture of telomeric chromatin revealed by correlative light and electron microscopy. Nucleic Acids Res..

[126] Soman A., Wong S.Y., Korolev N., Surya W., Lattmann S., Vogirala V.K. (2022). Columnar structure of human telomeric chromatin. Nature.

[127] Galati A., Rossetti L., Pisano S., Chapman L., Rhodes D., Savino M. (2006). The human telomeric protein TRF1 specifically recognizes nucleosomal binding sites and alters nucleosome structure. J. Mol. Biol..

[128] Pisano S., Leoni D., Galati A., Rhodes D., Savino M., Cacchione S. (2010). The human telomeric protein hTRF1 induces telomere-specific nucleosome mobility. Nucleic Acids Res..

[129] Hu H., van Roon A.M., Ghanim G.E., Ahsan B., Oluwole A.O., Peak-Chew S.Y. (2023). Structural basis of telomeric nucleosome recognition by shelterin factor TRF1. Sci. Adv..

[130] Wu Z.Q., Yang X., Weber G., Liu X. (2008). Plk1 phosphorylation of TRF1 is essential for its binding to telomeres. J. Biol. Chem..

[131] Zimmermann M., Kibe T., Kabir S., de Lange T. (2014). TRF1 negotiates TTAGGG repeat-associated replication problems by recruiting the BLM helicase and the TPP1/POT1 repressor of ATR signaling. Genes. Dev..

[132] Sfeir A., Kosiyatrakul S.T., Hockemeyer D., MacRae S.L., Karlseder J., Schildkraut C.L. (2009). Mammalian telomeres resemble fragile sites and require TRF1 for efficient replication. Cell.

[133] Tsutakawa S.E., Bacolla A., Katsonis P., Bralic A., Hamdan S.M., Lichtarge O. (2021). Decoding cancer variants of unknown significance for helicase-nuclease-RPA complexes orchestrating DNA repair during transcription and replication. Front Mol. Biosci..

[134] Bae N.S., Baumann P. (2007). A RAP1/TRF2 complex inhibits nonhomologous end-joining at human telomeric DNA ends. Mol. Cell.

[135] Ribes-Zamora A., Indiviglio S.M., Mihalek I., Williams C.L., Bertuch A.A. (2013). TRF2 interaction with Ku heterotetramerization interface gives insight into c-NHEJ prevention at human telomeres. Cell Rep..

[136] Baker A.M., Fu Q., Hayward W., Victoria S., Pedroso I.M., Lindsay S.M. (2011). The telomere binding protein TRF2 induces chromatin compaction. PLoS One.

[137] Wong S.Y., Soman A., Korolev N., Surya W., Chen Q., Shum W. (2024). The shelterin component TRF2 mediates columnar stacking of human telomeric chromatin. EMBO J..

[138] Deregowska A., Wnuk M. (2021). RAP1/TERF2IP-A multifunctional player in cancer development. Cancers (Basel).

[139] Mukherjee A.K., Sharma S., Sengupta S., Saha D., Kumar P., Hussain T. (2018). Telomere length-dependent transcription and epigenetic modifications in promoters remote from telomere ends. PLoS Genet..

[140] Peng J.C., Karpen G.H. (2008). Epigenetic regulation of heterochromatic DNA stability. Curr. Opin. Genet. Dev..

[141] Padeken J., Zeller P., Gasser S.M. (2015). Repeat DNA in genome organization and stability. Curr. Opin. Genet. Dev..

[142] Vader G., Blitzblau H.G., Tame M.A., Falk J.E., Curtin L., Hochwagen A. (2011). Protection of repetitive DNA borders from self-induced meiotic instability. Nature.

[143] Yang J., Li F. (2017). Are all repeats created equal? Understanding DNA repeats at an individual level. Curr. Genet..

[144] Henikoff S. (2000). Heterochromatin function in complex genomes. Biochim. Biophys. Acta.

[145] Peng J.C., Karpen G.H. (2007). H3K9 methylation and RNA interference regulate nucleolar organization and repeated DNA stability. Nat. Cell Biol..

[146] Alexiadis V., Ballestas M.E., Sanchez C., Winokur S., Vedanarayanan V., Warren M. (2007). RNAPol-ChIP analysis of transcription from FSHD-linked tandem repeats and satellite DNA. Biochim. Biophys. Acta.

[147] Enukashvily N.I., Donev R., Waisertreiger I.S., Podgornaya O.I. (2007). Human chromosome 1 satellite 3 DNA is decondensed, demethylated and transcribed in senescent cells and in A431 epithelial carcinoma cells. Cytogenet. Genome Res..

[148] Zhu Q., Pao G.M., Huynh A.M., Suh H., Tonnu N., Nederlof P.M. (2011). BRCA1 tumour suppression occurs via heterochromatin-mediated silencing. Nature.

[149] Bueno M.T.D., Baldascini M., Richard S., Lowndes N.F. (2018). Recruitment of lysine demethylase 2A to DNA double strand breaks and its interaction with 53BP1 ensures genome stability. Oncotarget.

[150] Ziv Y., Bielopolski D., Galanty Y., Lukas C., Taya Y., Schultz D.C. (2006). Chromatin relaxation in response to DNA double-strand breaks is modulated by a novel ATM- and KAP-1 dependent pathway. Nat. Cell Biol..

[151] Collins N., Poot R.A., Kukimoto I., Garcia-Jimenez C., Dellaire G., Varga-Weisz P.D. (2002). An ACF1-ISWI chromatin-remodeling complex is required for DNA replication through heterochromatin. Nat. Genet..

[152] Rowbotham S.P., Barki L., Neves-Costa A., Santos F., Dean W., Hawkes N. (2011). Maintenance of silent chromatin through replication requires SWI/SNF-like chromatin remodeler SMARCAD1. Mol. Cell.

[153] Chakraborty S., Pandita R.K., Hambarde S., Mattoo A.R., Charaka V., Ahmed K.M. (2018). SMARCAD1 phosphorylation and ubiquitination are required for resection during DNA double-strand break repair. iScience.

[154] Basta J., Rauchman M. (2015). The nucleosome remodeling and deacetylase complex in development and disease. Transl. Res..

[155] Ye Z., Xu S., Shi Y., Cheng X., Zhang Y., Roy S. (2024). GRB2 stabilizes RAD51 at reversed replication forks suppressing genomic instability and innate immunity against cancer. Nat. Commun..

[156] Marino-Ramirez L., Kann M.G., Shoemaker B.A., Landsman D. (2005). Histone structure and nucleosome stability. Expert Rev. Proteomics.

[157] Yi S.J., Kim K. (2018). Histone tail cleavage as a novel epigenetic regulatory mechanism for gene expression. BMB Rep..

[158] Kornberg R.D. (1999). Eukaryotic transcriptional control. Trends Cell Biol..

[159] MacAlpine D.M., Almouzni G. (2013). Chromatin and DNA replication. Cold Spring Harb Perspect. Biol..

[160] Kurat C.F., Yeeles J.T.P., Patel H., Early A., Diffley J.F.X. (2017). Chromatin controls DNA replication origin selection, lagging-strand synthesis, and replication fork rates. Mol. Cell.

[161] Yadav A.K., Polasek-Sedlackova H. (2024). Quantity and quality of minichromosome maintenance protein complexes couple replication licensing to genome integrity. Commun. Biol..

[162] Feng W., Chakraborty A. (2017). Fragility extraordinaire: unsolved mysteries of chromosome fragile sites. Adv. Exp. Med. Biol..

[163] Goehring L., Keegan S., Lahiri S., Xia W., Kong M., Jimenez-Sainz J. (2024). Dormant origin firing promotes head-on transcription-replication conflicts at transcription termination sites in response to BRCA2 deficiency. Nat. Commun..

[164] Uchino S., Ito Y., Sato Y., Handa T., Ohkawa Y., Tokunaga M. (2022). Live imaging of transcription sites using an elongating RNA polymerase II-specific probe. J. Cell. Biol..

[165] Meryet-Figuiere M., Alaei-Mahabadi B., Ali M.M., Mitra S., Subhash S., Pandey G.K. (2014). Temporal separation of replication and transcription during S-phase progression. Cell Cycle.

[166] Huvet M., Nicolay S., Touchon M., Audit B., d'Aubenton-Carafa Y., Arneodo A. (2007). Human gene organization driven by the coordination of replication and transcription. Genome Res..

[167] Prioleau M.N., MacAlpine D.M. (2016). DNA replication origins-where do we begin?. Genes. Dev..

[168] Zhao P.A., Sasaki T., Gilbert D.M. (2020). High-resolution Repli-Seq defines the temporal choreography of initiation, elongation and termination of replication in mammalian cells. Genome Biol..

[169] Venkatesh S., Workman J.L. (2015). Histone exchange, chromatin structure and the regulation of transcription. Nat. Rev. Mol. Cell Biol..

[170] Kaplan C.D., Laprade L., Winston F. (2003). Transcription elongation factors repress transcription initiation from cryptic sites. Science.

[171] Aguilera A., Garcia-Muse T. (2012). R loops: from transcription byproducts to threats to genome stability. Mol. Cell.

[172] Gaillard H., Aguilera A. (2016). Transcription as a threat to genome integrity. Annu. Rev. Biochem..

[173] Skourti-Stathaki K., Proudfoot N.J. (2014). A double-edged sword: R loops as threats to genome integrity and powerful regulators of gene expression. Genes. Dev..

[174] Helmrich A., Ballarino M., Nudler E., Tora L. (2013). Transcription-replication encounters, consequences and genomic instability. Nat. Struct. Mol. Biol..

[175] Sankar T.S., Wastuwidyaningtyas B.D., Dong Y., Lewis S.A., Wang J.D. (2016). The nature of mutations induced by replication-transcription collisions. Nature.

[176] Tubbs A., Nussenzweig A. (2017). Endogenous DNA damage as a source of genomic instability in cancer. Cell.

[177] Chatterjee N., Walker G.C. (2017). Mechanisms of DNA damage, repair, and mutagenesis. Environ. Mol. Mutagen.

[178] De Bont R., van Larebeke N. (2004). Endogenous DNA damage in humans: a review of quantitative data. Mutagenesis.

[179] Marnett L.J., Plastaras J.P. (2001). Endogenous DNA damage and mutation. Trends Genet..

[180] Jackson S.P., Bartek J. (2009). The DNA-damage response in human biology and disease. Nature.

[181] Ciccia A., Elledge S.J. (2010). The DNA damage response: making it safe to play with knives. Mol. Cell.

[182] Richardson C., Horikoshi N., Pandita T.K. (2004). The role of the DNA double-strand break response network in meiosis. DNA Repair (Amst).

[183] Du F., Zhang M., Li X., Yang C., Meng H., Wang D. (2014). Dimer monomer transition and dimer re-formation play important role for ATM cellular function during DNA repair. Biochem. Biophys. Res. Commun..

[184] Bakkenist C.J., Kastan M.B. (2003). DNA damage activates ATM through intermolecular autophosphorylation and dimer dissociation. Nature.

[185] Scott S.P., Pandita T.K. (2006). The cellular control of DNA double-strand breaks. J. Cell Biochem..

[186] Udayakumar D., Horikoshi N., Mishra L., Hunt C., Pandita T.K. (2015). Detecting ATM-dependent chromatin modification in DNA damage response. Methods Mol. Biol..

[187] Kumar R., Horikoshi N., Singh M., Gupta A., Misra H.S., Albuquerque K. (2012). Chromatin modifications and the DNA damage response to ionizing radiation. Front Oncol.

[188] Mir U.S., Bhat A., Mushtaq A., Pandita S., Altaf M., Pandita T.K. (2021). Role of histone acetyltransferases MOF and Tip60 in genome stability. DNA Repair (Amst).

[189] Mushtaq A., Mir U.S., Hunt C.R., Pandita S., Tantray W.W., Bhat A. (2021). Role of histone methylation in maintenance of genome integrity. Genes (Basel).

[190] Ye Z., Xu S., Shi Y., Bacolla A., Syed A., Moiani D. (2021). GRB2 enforces homology-directed repair initiation by MRE11. Sci. Adv..

[191] Gupta A., Sharma G.G., Young C.S., Agarwal M., Smith E.R., Paull T.T. (2005). Involvement of human MOF in ATM function. Mol. Cell Biol..

[192] Sharma G.G., So S., Gupta A., Kumar R., Cayrou C., Avvakumov N. (2010). MOF and histone H4 acetylation at lysine 16 are critical for DNA damage response and double-strand break repair. Mol. Cell Biol..

[193] Swift M.L., Zhou R., Syed A., Moreau L.A., Tomasik B., Tainer J.A. (2023). Dynamics of the DYNLL1-MRE11 complex regulate DNA end resection and recruitment of Shieldin to DSBs. Nat. Struct. Mol. Biol..

[194] Gupta A., Hunt C.R., Hegde M.L., Chakraborty S., Chakraborty S., Udayakumar D. (2014). MOF phosphorylation by ATM regulates 53BP1-mediated double-strand break repair pathway choice. Cell Rep..

[195] Singh M., Bacolla A., Chaudhary S., Hunt C.R., Pandita S., Chauhan R. (2020). Histone acetyltransferase MOF orchestrates outcomes at the crossroad of oncogenesis, DNA damage response, proliferation, and stem cell development. Mol. Cell Biol..

[196] Jacquet K., Fradet-Turcotte A., Avvakumov N., Lambert J.P., Roques C., Pandita R.K. (2016). The TIP60 complex regulates bivalent chromatin recognition by 53BP1 through direct H4K20me binding and H2AK15 acetylation. Mol. Cell.

[197] Hunt C.R., Pandita T.K. (2019). "What's Past is Prologue": pre-existing epigenetic transcriptional marks may also influence DNA repair pathway choice. Radiat. Res..

[198] Lindahl T., Barnes D.E. (2000). Repair of endogenous DNA damage. Cold Spring Harb Symp. Quant. Biol..

[199] Scharer O.D. (2013). Nucleotide excision repair in eukaryotes. Cold Spring Harb Perspect. Biol..

[200] Eckelmann B.J., Bacolla A., Wang H., Ye Z., Guerrero E.N., Jiang W. (2020). XRCC1 promotes replication restart, nascent fork degradation and mutagenic DNA repair in BRCA2-deficient cells. NAR Cancer.

[201] Lieber M.R. (2010). The mechanism of double-strand DNA break repair by the nonhomologous DNA end-joining pathway. Annu. Rev. Biochem..

[202] Heyer W.D., Ehmsen K.T., Liu J. (2010). Regulation of homologous recombination in eukaryotes. Annu. Rev. Genet..

[203] Syed A., Tainer J.A. (2018). The MRE11-RAD50-NBS1 complex conducts the orchestration of damage signaling and outcomes to stress in DNA replication and repair. Annu. Rev. Biochem..

[204] Green C.M., Almouzni G. (2002). When repair meets chromatin. First in series on chromatin dynamics. EMBO Rep..

[205] Gong F., Kwon Y., Smerdon M.J. (2005). Nucleotide excision repair in chromatin and the right of entry. DNA Repair (Amst).

[206] Schuster-Bockler B., Lehner B. (2012). Chromatin organization is a major influence on regional mutation rates in human cancer cells. Nature.

[207] Zheng C.L., Wang N.J., Chung J., Moslehi H., Sanborn J.Z., Hur J.S. (2014). Transcription restores DNA repair to heterochromatin, determining regional mutation rates in cancer genomes. Cell Rep..

[208] Misteli T. (2007). Beyond the sequence: cellular organization of genome function. Cell.

[209] Supek F., Lehner B. (2015). Differential DNA mismatch repair underlies mutation rate variation across the human genome. Nature.

[210] Adam S., Dabin J., Polo S.E. (2015). Chromatin plasticity in response to DNA damage: the shape of things to come. DNA Repair (Amst).

[211] Timinszky G., Till S., Hassa P.O., Hothorn M., Kustatscher G., Nijmeijer B. (2009). A macrodomain-containing histone rearranges chromatin upon sensing PARP1 activation. Nat. Struct. Mol. Biol..

[212] Rogakou E.P., Pilch D.R., Orr A.H., Ivanova V.S., Bonner W.M. (1998). DNA double-stranded breaks induce histone H2AX phosphorylation on serine 139. J. Biol. Chem..

[213] Cowell I.G., Sunter N.J., Singh P.B., Austin C.A., Durkacz B.W., Tilby M.J. (2007). gammaH2AX foci form preferentially in euchromatin after ionising-radiation. PLoS One.

[214] Kim J.A., Kruhlak M., Dotiwala F., Nussenzweig A., Haber J.E. (2007). Heterochromatin is refractory to gamma-H2AX modification in yeast and mammals. J. Cell Biol..

[215] Sabarinathan R., Mularoni L., Deu-Pons J., Gonzalez-Perez A., Lopez-Bigas N. (2016). Nucleotide excision repair is impaired by binding of transcription factors to DNA. Nature.

[216] Perera D., Poulos R.C., Shah A., Beck D., Pimanda J.E., Wong J.W. (2016). Differential DNA repair underlies mutation hotspots at active promoters in cancer genomes. Nature.

[217] Pleasance E.D., Cheetham R.K., Stephens P.J., McBride D.J., Humphray S.J., Greenman C.D. (2010). A comprehensive catalogue of somatic mutations from a human cancer genome. Nature.

[218] Bralic A., Tehseen M., Sobhy M.A., Tsai C.L., Alhudhali L., Yi G. (2023). A scanning-to-incision switch in TFIIH-XPG induced by DNA damage licenses nucleotide excision repair. Nucleic Acids Res..

[219] Yu J., Yan C., Paul T., Brewer L., Tsutakawa S.E., Tsai C.L. (2024). Molecular architecture and functional dynamics of the pre-incision complex in nucleotide excision repair. Nat. Commun..

[220] Sugasawa K., Masutani C., Hanaoka F. (1993). Cell-free repair of UV-damaged simian virus 40 chromosomes in human cell extracts. I. Development of a cell-free system detecting excision repair of UV-irradiated SV40 chromosomes. J. Biol. Chem..

[221] Araki M., Masutani C., Maekawa T., Watanabe Y., Yamada A., Kusumoto R. (2000). Reconstitution of damage DNA excision reaction from SV40 minichromosomes with purified nucleotide excision repair proteins. Mutat. Res..

[222] Mitchell D.L., Nguyen T.D., Cleaver J.E. (1990). Nonrandom induction of pyrimidine-pyrimidone (6-4) photoproducts in ultraviolet-irradiated human chromatin. J. Biol. Chem..

[223] Mellon I., Spivak G., Hanawalt P.C. (1987). Selective removal of transcription-blocking DNA damage from the transcribed strand of the mammalian DHFR gene. Cell.

[224] San Filippo J., Sung P., Klein H. (2008). Mechanism of eukaryotic homologous recombination. Annu. Rev. Biochem..

[225] Shibata A., Moiani D., Arvai A.S., Perry J., Harding S.M., Genois M.M. (2014). DNA double-strand break repair pathway choice is directed by distinct MRE11 nuclease activities. Mol. Cell.

[226] Bekker-Jensen S., Mailand N. (2010). Assembly and function of DNA double-strand break repair foci in mammalian cells. DNA Repair (Amst).

[227] Kinner A., Wu W., Staudt C., Iliakis G. (2008). Gamma-H2AX in recognition and signaling of DNA double-strand breaks in the context of chromatin. Nucleic Acids Res..

[228] Aleksandrov R., Hristova R., Stoynov S., Gospodinov A. (2020). The chromatin response to double-strand DNA breaks and their repair. Cells.

[229] Goodarzi A.A., Kurka T., Jeggo P.A. (2011). KAP-1 phosphorylation regulates CHD3 nucleosome remodeling during the DNA double-strand break response. Nat. Struct. Mol. Biol..

[230] Goodarzi A.A., Noon A.T., Deckbar D., Ziv Y., Shiloh Y., Lobrich M. (2008). ATM signaling facilitates repair of DNA double-strand breaks associated with heterochromatin. Mol. Cell.

[231] Murga M., Jaco I., Fan Y., Soria R., Martinez-Pastor B., Cuadrado M. (2007). Global chromatin compaction limits the strength of the DNA damage response. J. Cell Biol..

[232] Duan M.R., Smerdon M.J. (2010). UV damage in DNA promotes nucleosome unwrapping. J. Biol. Chem..

[233] Muftuoglu M., Selzer R., Tuo J., Brosh R.M., Bohr V.A. (2002). Phenotypic consequences of mutations in the conserved motifs of the putative helicase domain of the human Cockayne syndrome group B gene. Gene.

[234] Citterio E., Rademakers S., van der Horst G.T., van Gool A.J., Hoeijmakers J.H., Vermeulen W. (1998). Biochemical and biological characterization of wild-type and ATPase-deficient Cockayne syndrome B repair protein. J. Biol. Chem..

[235] Selzer R.R., Nyaga S., Tuo J., May A., Muftuoglu M., Christiansen M. (2002). Differential requirement for the ATPase domain of the Cockayne syndrome group B gene in the processing of UV-induced DNA damage and 8-oxoguanine lesions in human cells. Nucleic Acids Res..

[236] Lake R.J., Geyko A., Hemashettar G., Zhao Y., Fan H.Y. (2010). UV-induced association of the CSB remodeling protein with chromatin requires ATP-dependent relief of N-terminal autorepression. Mol. Cell.

[237] Citterio E., Van Den Boom V., Schnitzler G., Kanaar R., Bonte E., Kingston R.E. (2000). ATP-dependent chromatin remodeling by the Cockayne syndrome B DNA repair-transcription-coupling factor. Mol. Cell Biol..

[238] Kruhlak M.J., Celeste A., Dellaire G., Fernandez-Capetillo O., Muller W.G., McNally J.G. (2006). Changes in chromatin structure and mobility in living cells at sites of DNA double-strand breaks. J. Cell Biol..

[239] Podhorecka M., Skladanowski A., Bozko P. (2010). H2AX phosphorylation: its role in DNA damage response and cancer therapy. J. Nucleic Acids.

[240] Price B.D., D'Andrea A.D. (2013). Chromatin remodeling at DNA double-strand breaks. Cell.

[241] Luijsterburg M.S., Lindh M., Acs K., Vrouwe M.G., Pines A., van Attikum H. (2012). DDB2 promotes chromatin decondensation at UV-induced DNA damage. J. Cell Biol..

[242] Strickfaden H., McDonald D., Kruhlak M.J., Haince J.F., Th'ng J.P.H., Rouleau M. (2016). Poly(ADP-ribosyl)ation-dependent transient chromatin decondensation and histone displacement following laser microirradiation. J. Biol. Chem..

[243] Cadet J., Douki T., Ravanat J.L., Di Mascio P. (2009). Sensitized formation of oxidatively generated damage to cellular DNA by UVA radiation. Photochem. Photobiol. Sci..

[244] Greinert R., Volkmer B., Henning S., Breitbart E.W., Greulich K.O., Cardoso M.C. (2012). UVA-induced DNA double-strand breaks result from the repair of clustered oxidative DNA damages. Nucleic Acids Res..

[245] Berkovich E., Monnat R.J., Kastan M.B. (2007). Roles of ATM and NBS1 in chromatin structure modulation and DNA double-strand break repair. Nat. Cell Biol..

[246] Courilleau C., Chailleux C., Jauneau A., Grimal F., Briois S., Boutet-Robinet E. (2012). The chromatin remodeler p400 ATPase facilitates Rad51-mediated repair of DNA double-strand breaks. J. Cell Biol..

[247] Hauer M.H., Seeber A., Singh V., Thierry R., Sack R., Amitai A. (2017). Histone degradation in response to DNA damage enhances chromatin dynamics and recombination rates. Nat. Struct. Mol. Biol..

[248] Maddi K., Sam D.K., Bonn F., Prgomet S., Tulowetzke E., Akutsu M. (2020). Wss1 promotes replication stress tolerance by degrading histones. Cell Rep..

[249] Fierz B., Chatterjee C., McGinty R.K., Bar-Dagan M., Raleigh D.P., Muir T.W. (2011). Histone H2B ubiquitylation disrupts local and higher-order chromatin compaction. Nat. Chem. Biol..

[250] Moyal L., Lerenthal Y., Gana-Weisz M., Mass G., So S., Wang S.Y. (2011). Requirement of ATM-dependent monoubiquitylation of histone H2B for timely repair of DNA double-strand breaks. Mol. Cell.

[251] Oliveira D.V., Kato A., Nakamura K., Ikura T., Okada M., Kobayashi J. (2014). Histone chaperone FACT regulates homologous recombination by chromatin remodeling through interaction with RNF20. J. Cell Sci..

[252] Klement K., Luijsterburg M.S., Pinder J.B., Cena C.S., Del Nero V., Wintersinger C.M. (2014). Opposing ISWI- and CHD-class chromatin remodeling activities orchestrate heterochromatic DNA repair. J. Cell Biol..

[253] Wang H., Zhai L., Xu J., Joo H.Y., Jackson S., Erdjument-Bromage H. (2006). Histone H3 and H4 ubiquitylation by the CUL4-DDB-ROC1 ubiquitin ligase facilitates cellular response to DNA damage. Mol. Cell.

[254] Smith R., Sellou H., Chapuis C., Huet S., Timinszky G. (2018). CHD3 and CHD4 recruitment and chromatin remodeling activity at DNA breaks is promoted by early poly(ADP-ribose)-dependent chromatin relaxation. Nucleic Acids Res..

[255] Yang G., Chen Y., Wu J., Chen S.H., Liu X., Singh A.K. (2020). Poly(ADP-ribosyl)ation mediates early phase histone eviction at DNA lesions. Nucleic Acids Res..

[256] Leidecker O., Bonfiglio J.J., Colby T., Zhang Q., Atanassov I., Zaja R. (2016). Serine is a new target residue for endogenous ADP-ribosylation on histones. Nat. Chem. Biol..

[257] Morales J., Li L., Fattah F.J., Dong Y., Bey E.A., Patel M. (2014). Review of poly (ADP-ribose) polymerase (PARP) mechanisms of action and rationale for targeting in cancer and other diseases. Crit. Rev. Eukaryot. Gene Expr..

[258] Houl J.H., Ye Z., Brosey C.A., Balapiti-Modarage L.P.F., Namjoshi S., Bacolla A. (2019). Selective small molecule PARG inhibitor causes replication fork stalling and cancer cell death. Nat. Commun..

[259] Bonner W.M., Redon C.E., Dickey J.S., Nakamura A.J., Sedelnikova O.A., Solier S. (2008). GammaH2AX and cancer. Nat. Rev. Cancer.

[260] Prabhu K.S., Kuttikrishnan S., Ahmad N., Habeeba U., Mariyam Z., Suleman M. (2024). H2AX: a key player in DNA damage response and a promising target for cancer therapy. Biomed. Pharmacother..

[261] Aquila L., Atanassov B.S. (2020). Regulation of histone ubiquitination in response to DNA double strand breaks. Cells.

[262] Thorslund T., Ripplinger A., Hoffmann S., Wild T., Uckelmann M., Villumsen B. (2015). Histone H1 couples initiation and amplification of ubiquitin signalling after DNA damage. Nature.

[263] Mattiroli F., Vissers J.H., van Dijk W.J., Ikpa P., Citterio E., Vermeulen W. (2012). RNF168 ubiquitinates K13-15 on H2A/H2AX to drive DNA damage signaling. Cell.

[264] Gatti M., Pinato S., Maiolica A., Rocchio F., Prato M.G., Aebersold R. (2015). RNF168 promotes noncanonical K27 ubiquitination to signal DNA damage. Cell Rep..

[265] Densham R.M., Garvin A.J., Stone H.R., Strachan J., Baldock R.A., Daza-Martin M. (2016). Human BRCA1-BARD1 ubiquitin ligase activity counteracts chromatin barriers to DNA resection. Nat. Struct. Mol. Biol..

[266] Clouaire T., Rocher V., Lashgari A., Arnould C., Aguirrebengoa M., Biernacka A. (2018). Comprehensive mapping of histone modifications at DNA double-strand breaks deciphers repair pathway chromatin signatures. Mol Cell.

[267] Li X., Tyler J.K. (2016). Nucleosome disassembly during human non-homologous end joining followed by concerted HIRA- and CAF-1-dependent reassembly. Elife.

[268] Polo S.E., Roche D., Almouzni G. (2006). New histone incorporation marks sites of UV repair in human cells. Cell.

[269] Adam S., Dabin J., Bai S.K., Polo S.E. (2015). Imaging local deposition of newly synthesized histones in UVC-damaged chromatin. Methods Mol. Biol..

[270] Shanbhag N.M., Rafalska-Metcalf I.U., Balane-Bolivar C., Janicki S.M., Greenberg R.A. (2010). ATM-dependent chromatin changes silence transcription in cis to DNA double-strand breaks. Cell..

[271] Adam S., Polo S.E., Almouzni G. (2013). Transcription recovery after DNA damage requires chromatin priming by the H3.3 histone chaperone HIRA. Cell.

[272] Shi L., Wen H., Shi X. (2017). The histone variant H3.3 in transcriptional regulation and human disease. J. Mol. Biol..

[273] Roemer A., Mohammed L., Strickfaden H., Underhill D.A., Hendzel M.J. (2022). Mechanisms governing the accessibility of DNA damage proteins to constitutive heterochromatin. Front. Genet..

[274] Hauer M.H., Gasser S.M. (2017). Chromatin and nucleosome dynamics in DNA damage and repair. Genes. Dev..

[275] Dabin J., Mori M., Polo S.E. (2023). The DNA damage response in the chromatin context: a coordinated process. Curr. Opin. Cell Biol..

[276] Takata H., Hanafusa T., Mori T., Shimura M., Iida Y., Ishikawa K. (2013). Chromatin compaction protects genomic DNA from radiation damage. PLoS One.

[277] Janssen A., Breuer G.A., Brinkman E.K., van der Meulen A.I., Borden S.V., van Steensel B. (2016). A single double-strand break system reveals repair dynamics and mechanisms in heterochromatin and euchromatin. Genes. Dev..

[278] Harding S.M., Boiarsky J.A., Greenberg R.A. (2015). ATM dependent silencing links nucleolar chromatin reorganization to DNA damage recognition. Cell Rep..

[279] van Sluis M., McStay B. (2015). A localized nucleolar DNA damage response facilitates recruitment of the homology-directed repair machinery independent of cell cycle stage. Genes Dev..

[280] Fortuny A., Polo S.E. (2018). The response to DNA damage in heterochromatin domains. Chromosoma.

[281] Han C., Srivastava A.K., Cui T., Wang Q.E., Wani A.A. (2016). Differential DNA lesion formation and repair in heterochromatin and euchromatin. Carcinogenesis.

[282] Qian J., Zhou X., Tanaka K., Takahashi A. (2023). Alteration in the chromatin landscape during the DNA damage response: continuous rotation of the gear driving cellular senescence and aging. DNA Repair (Amst).

[283] Woodbine L., Brunton H., Goodarzi A.A., Shibata A., Jeggo P.A. (2011). Endogenously induced DNA double strand breaks arise in heterochromatic DNA regions and require ataxia telangiectasia mutated and Artemis for their repair. Nucleic Acids Res..

[284] Schmid W., Vischer D. (1969). Spontaneous fragility of an abnormally wide secondary constriction region in a human chromosome no. 9. Humangenetik.

[285] Li X.Z., Reidy J.A., Wheeler V.A., Chen A.T. (1986). Folic acid and chromosome breakage. III. Types and frequencies of spontaneous chromosome aberrations in proliferating lymphocytes. Mutat. Res..

[286] Arlt M.F., Casper A.M., Glover T.W. (2003). Common fragile sites. Cytogenet. Genome. Res..

[287] Cleary J.D., Pearson C.E. (2003). The contribution of cis-elements to disease-associated repeat instability: clinical and experimental evidence. Cytogenet. Genome. Res..

[288] Glover T.W., Berger C., Coyle J., Echo B. (1984). DNA polymerase alpha inhibition by aphidicolin induces gaps and breaks at common fragile sites in human chromosomes. Hum. Genet..

[289] Schmid M., Feichtinger W., Jessberger A., Kohler J., Lange R. (1986). The fragile site (16) (q22). I. Induction by AT-specific DNA-ligands and population frequency. Hum. Genet..

[290] Hecht F., Sutherland G.R. (1984). Detection of the fragile X chromosome and other fragile sites. Clin. Genet..

[291] Sutherland G.R., Parslow M.I., Baker E. (1985). New classes of common fragile sites induced by 5-azacytidine and bromodeoxyuridine. Hum. Genet..

[292] Irony-Tur Sinai M., Kerem B. (2019). Genomic instability in fragile sites-still adding the pieces. Genes Chromosomes Cancer.

[293] Lukusa T., Fryns J.P. (2008). Human chromosome fragility. Biochim. Biophys. Acta.

[294] Yu S., Mangelsdorf M., Hewett D., Hobson L., Baker E., Eyre H.J. (1997). Human chromosomal fragile site FRA16B is an amplified AT-rich minisatellite repeat. Cell.

[295] Sarni D., Kerem B. (2016). The complex nature of fragile site plasticity and its importance in cancer. Curr. Opin. Cell Biol..

[296] Glover T.W., Wilson T.E., Arlt M.F. (2017). Fragile sites in cancer: more than meets the eye. Nat. Rev. Cancer.

[297] Kaushal S., Freudenreich C.H. (2019). The role of fork stalling and DNA structures in causing chromosome fragility. Genes Chromosomes Cancer.

[298] Mishmar D., Rahat A., Scherer S.W., Nyakatura G., Hinzmann B., Kohwi Y. (1998). Molecular characterization of a common fragile site (FRA7H) on human chromosome 7 by the cloning of a simian virus 40 integration site. Proc. Natl. Acad. Sci. U. S. A..

[299] Sarai A., Mazur J., Nussinov R., Jernigan R.L. (1989). Sequence dependence of DNA conformational flexibility. Biochemistry.

[300] Zlotorynski E., Rahat A., Skaug J., Ben-Porat N., Ozeri E., Hershberg R. (2003). Molecular basis for expression of common and rare fragile sites. Mol. Cell Biol..

[301] Ragland R.L., Glynn M.W., Arlt M.F., Glover T.W. (2008). Stably transfected common fragile site sequences exhibit instability at ectopic sites. Genes Chromosomes Cancer.

[302] Mitsui J., Takahashi Y., Goto J., Tomiyama H., Ishikawa S., Yoshino H. (2010). Mechanisms of genomic instabilities underlying two common fragile-site-associated loci, PARK2 and DMD, in germ cell and cancer cell lines. Am. J. Hum. Genet..

[303] Debacker K., Winnepenninckx B., Ben-Porat N., FitzPatrick D., Van Luijk R., Scheers S. (2007). FRA18C: a new aphidicolin-inducible fragile site on chromosome 18q22, possibly associated with in vivo chromosome breakage. J. Med. Genet..

[304] Tubbs A., Sridharan S., van Wietmarschen N., Maman Y., Callen E., Stanlie A. (2018). Dual roles of poly(dA:dT) tracts in replication initiation and fork collapse. Cell.

[305] Savelyeva L., Brueckner L.M. (2014). Molecular characterization of common fragile sites as a strategy to discover cancer susceptibility genes. Cell Mol. Life Sci..

[306] Bowen B.C. (1981). DNA fragments associated with chromosome scaffolds. Nucleic Acids Res..

[307] Garribba L., Vogel I., Lerdrup M., Goncalves Dinis M.M., Ren L., Liu Y. (2021). Folate deficiency triggers the abnormal segregation of a region with large cluster of CG-rich trinucleotide repeats on human chromosome 2. Front. Genet..

[308] Hambarde S., Tsai C.L., Pandita R.K., Bacolla A., Maitra A., Charaka V. (2021). EXO5-DNA structure and BLM interactions direct DNA resection critical for ATR-dependent replication restart. Mol. Cell.

[309] Pichierri P., Ammazzalorso F., Bignami M., Franchitto A. (2011). The Werner syndrome protein: linking the replication checkpoint response to genome stability. Aging (Albany NY).

[310] Perry J.J., Yannone S.M., Holden L.G., Hitomi C., Asaithamby A., Han S. (2006). WRN exonuclease structure and molecular mechanism imply an editing role in DNA end processing. Nat. Struct. Mol. Biol..

[311] Pirzio L.M., Pichierri P., Bignami M., Franchitto A. (2008). Werner syndrome helicase activity is essential in maintaining fragile site stability. J. Cell Biol..

[312] Tuduri S., Crabbe L., Conti C., Tourriere H., Holtgreve-Grez H., Jauch A. (2009). Topoisomerase I suppresses genomic instability by preventing interference between replication and transcription. Nat. Cell Biol..

[313] Sartori A.A., Lukas C., Coates J., Mistrik M., Fu S., Bartek J. (2007). Human CtIP promotes DNA end resection. Nature.

[314] Longo M.A., Roy S., Chen Y., Tomaszowski K.H., Arvai A.S., Pepper J.T. (2023). RAD51C-XRCC3 structure and cancer patient mutations define DNA replication roles. Nat. Commun..

[315] Howlett N.G., Taniguchi T., Durkin S.G., D'Andrea A.D., Glover T.W. (2005). The Fanconi anemia pathway is required for the DNA replication stress response and for the regulation of common fragile site stability. Hum. Mol. Genet..

[316] Bournique E., Dall'Osto M., Hoffmann J.S., Bergoglio V. (2018). Role of specialized DNA polymerases in the limitation of replicative stress and DNA damage transmission. Mutat. Res..

[317] Walsh E., Wang X., Lee M.Y., Eckert K.A. (2013). Mechanism of replicative DNA polymerase delta pausing and a potential role for DNA polymerase kappa in common fragile site replication. J. Mol. Biol..

[318] Bergoglio V., Boyer A.S., Walsh E., Naim V., Legube G., Lee M.Y. (2013). DNA synthesis by Pol eta promotes fragile site stability by preventing under-replicated DNA in mitosis. J. Cell Biol..

[319] Noel T., Hessel V. (2013). Membrane microreactors: gas-liquid reactions made easy. ChemSusChem.

[320] Bhat A., Andersen P.L., Qin Z., Xiao W. (2013). Rev3, the catalytic subunit of Polzeta, is required for maintaining fragile site stability in human cells. Nucleic Acids Res.

[321] Twayana S., Bacolla A., Barreto-Galvez A., De-Paula R.B., Drosopoulos W.C., Kosiyatrakul S.T. (2021). Translesion polymerase eta both facilitates DNA replication and promotes increased human genetic variation at common fragile sites. Proc. Natl. Acad. Sci. U. S. A..

